# Unraveling the relationships between alpha- and beta-adrenergic modulation and the risk of heart failure

**DOI:** 10.3389/fcvm.2023.1148931

**Published:** 2023-10-18

**Authors:** Claire Baudier, Françoise Fougerousse, Folkert W. Asselbergs, Mickael Guedj, Michel Komajda, Dipak Kotecha, R. Thomas Lumbers, Amand F. Schmidt, Benoît Tyl

**Affiliations:** ^1^ Translational Medicine Division, Institut de Recherches Internationales Servier, Suresnes, France; ^2^ Center for Therapeutic Innovation Cardiovascular & Metabolic Disease, Institut de Recherches Internationales Servier, Suresnes, France; ^3^ Institute of Health Informatics, University College London, London, United Kingdom; ^4^ The National Institute for Health Research University College London Hospitals Biomedical Research Centre, University College London, London, United Kingdom; ^5^ Department of Cardiology, Amsterdam Cardiovascular Sciences, Amsterdam University Medical Centre, University of Amsterdam, Amsterdam, Netherlands; ^6^ Department of Cardiology, Hospital Saint Joseph and Sorbonne University, Paris, France; ^7^ Institute of Cardiovascular Sciences, University of Birmingham, Birmingham, United Kingdom; ^8^ West Midlands NHS Secure Data Environment, University Hospitals Birmingham NHS Foundation Trust, Birmingham, United Kingdom; ^9^ NIHR Birmingham Biomedical Research Centre, Birmingham, United Kingdom; ^10^ Health Data Research UK London, University College London, London, United Kingdom; ^11^ UCL British Heart Foundation Research Accelerator, London, United Kingdom; ^12^ Institute of Cardiovascular Science, Faculty of Population Health Sciences, University College London, London, United Kingdom; ^13^ Department of Cardiology, Division Heart and Lungs, University Medical Center Utrecht, Utrecht University, Utrecht, Netherlands

**Keywords:** Mendelian randomization, adrenergic receptors, beta-blockers, alpha-blockers, target validation, drug

## Abstract

**Background:**

The effects of α and ß adrenergic receptor modulation on the risk of developing heart failure (HF) remains uncertain due to a lack of randomized controlled trials. This study aimed to estimate the effects of α and ß adrenergic receptors modulation on the risk of HF and to provide proof of principle for genetic target validation studies in HF.

**Methods:**

Genetic variants within the cis regions encoding the adrenergic receptors α1A, α2B, ß1, and ß2 associated with blood pressure in a 757,601-participant genome-wide association study (GWAS) were selected as instruments to perform a drug target Mendelian randomization study. Effects of these variants on HF risk were derived from the HERMES GWAS (542,362 controls; 40,805 HF cases).

**Results:**

Lower α1A or ß1 activity was associated with reduced HF risk: odds ratio (OR) 0.83 (95% CI 0.74–0.93, *P* = 0.001) and 0.95 (95% CI 0.93–0.97, *P* = 8 × 10^−6^). Conversely, lower α2B activity was associated with increased HF risk: OR 1.09 (95% CI 1.05–1.12, *P* = 3 × 10^−7^). No evidence of an effect of lower ß2 activity on HF risk was found: OR 0.99 (95% CI 0.92–1.07, *P* = 0.95). Complementary analyses showed that these effects were consistent with those on left ventricular dimensions and acted independently of any potential effect on coronary artery disease.

**Conclusions:**

This study provides genetic evidence that α1A or ß1 receptor inhibition will likely decrease HF risk, while lower α2B activity may increase this risk. Genetic variant analysis can assist with drug development for HF prevention.

## 1. Introduction

Heart Failure's (HF) prognosis is worse than that of most cancers despite significant progress in disease management (1). However, the willingness of drug developers to launch new developments has been hindered by the rising costs and high failure rate of clinical studies (2). This calls for methods that can assist in the selection of drug targets, and hence improve the chance of success of clinical trials.

Several adaptive changes in HF are mediated by an over-activation of the sympathetic nervous system as evidenced in HF with reduced or mildly-reduced left ventricular ejection fraction (LVEF) (3, 4). The family of adrenergic receptors involved in its regulation includes 9 different subtypes: three α1-receptors (A, B and D), three α2-receptors (A, B and C), and three ß-adrenergic receptors (ß1, ß2, and ß3) (3). Various specific and/or non-specific inhibitors or activators have been developed and tested in HF. However, while the benefit of ß1-blockade has been well established (4), little is known about the effect of the modulation of other adrenergic receptors. Most available data are observational or from small-scale randomized clinical trials, and their interpretation remains controversial, especially for α-blockers shown either to be detrimental (5), or protective from HF (3, 6). Furthermore, whilst ß1 antagonism is guideline-recommended in HF with reduced LVEF (4), evidence for a role in HF prevention is not as well established (7).

Many traditional candidate gene studies have assessed the effect of adrenoreceptor modulation on HF, but their results are controversial or inconclusive (8). On the contrary, Mendelian randomization (MR) studies based on well-powered Genome-Wide Association studies (GWAS), are a powerful way to predict the probability of success of drug development as they leverage the natural randomization of genetic variants at conception to mimic the design of randomized clinical trials. Drug target MRs have recently been proposed as an adaptation of the classic MR design to specifically assess the effect of the modulation of a drug target, rather than a biomarker, on a disease by using genetic variants related to the function or expression of the drug target protein as instrumental variables (9).

We aimed to decipher the role of the various adrenoreceptors in HF by using a drug target MR to estimate the effects of their inhibition on the risk of developing HF and the left ventricular (LV) dimensions, and to provide proof of principle for genetic target validation studies in HF to prioritize novel therapeutic approaches. For targets causally related to HF, we also studied their effect on the risk of coronary artery disease (CAD) to gain further insight into the mechanisms involved.

## 2. Materials and methods

### 2.1. Study design

Drug target validation MR studies follow the same principle as classical MRs that assess the causal relationship between an exposure (e.g., biomarker) and an outcome (e.g., disease risk), but restricted the genetic variants selection to the cis-region of the gene encoding the drug target of interest (exposure) to build the genetic instrument rather than selecting them from across the genome (Supplementary Figure S1) (9).

We used relevant downstream traits: blood pressure (BP) and heart rate (the main cardiovascular biomarkers affected by sympathetic nervous system modulation), as proxies for receptor activity, to select the cis-variants and weight their effect (Supplementary Figure S1) (9). As trait-associated variants are frequently associated with gene expression, we performed additional MR analyses with variants modulating adrenoreceptor expression or their protein concentration in blood, when available.

### 2.2. Data sources

#### 2.2.1. Data sources used to build the genetic instruments

A list and a description of the GWAS summary statistics used are provided in Supplementary Table S1. Genetic association estimates for diastolic and systolic blood pressure (BP) were obtained from a GWAS meta-analysis of 757,601 individuals with European ancestry drawn from the UK Biobank (10) and the International Consortium of BP GWAS meta-analysis (11). Genetic association estimates for resting heart rate were obtained from a GWAS of 458,969 individuals with European ancestry drawn from the UK Biobank, where association analysis was adjusted for age, sex, smoking, genotyping array, and 20 ancestry principal components (12).

Expression quantitative trait loci (eQTL) were obtained from the Genotype-Tissue Expression (GTEx) portal (release version 8) that includes 15,201 RNA-sequencing samples from 54 non-diseased tissues of 838 postmortem donors (85.3% European American, 66.4% male) (13). As 49 tissue types are included in the eQTL analyses from GTEx portal, we restricted the selection for our study to the eQTLs data from the heart's left ventricle (LV).

Protein quantitative trait loci (pQTL) data were obtained from a cohort of 3,301 participants of European descent from the INTERVAL study that includes about 50,000 healthy participants nested within a randomized trial of varying blood donation intervals (14). The relative concentrations of 3,622 blood proteins or protein complexes were assessed for each donor by modified aptamers.

#### 2.2.2. Outcome data sources

The Heart Failure (HF) risk GWAS comprising 40,805 HF cases and 542,362 controls was derived from a GWAS meta-analysis of HF of the HERMES consortium of European ancestry, which includes 68,157 HF cases and 949,888 controls (15). The two-sample MR study design used for our analysis requires avoiding an important overlap of participants between the exposure and outcome GWAS. As the BP and heart rate GWAS we used included a large proportion of subjects from the UK Biobank cohort, the genetic association estimates for HF risk were obtained from the HERMES GWAS after the exclusion of the UK Biobank participants. Cases included participants with a clinical diagnosis of HF of any etiology with no inclusion criteria based on left ventricular ejection fraction (LVEF); controls were participants without HF (16). All studies of this meta-analysis included age and sex (except for single-sex studies) as covariates in the regression models. Principal components were included as covariates for individual studies as appropriate. This same GWAS derived from HERMES was also adjusted for CAD risk using Multi-trait Conditional and Joint Analysis (mtCOJO) (17) to obtain a second GWAS used to explore the mediation of HF risk through CAD.

Genetic association estimates for the LV dimensions were obtained from GWAS of cardiac magnetic resonance imaging (MRI)-derived LV measurements drawn from the UK Biobank: Left ventricular mass (LV mass), Left ventricular end-diastolic volume (LVEDV), Left ventricular end-systolic volume (LVESV), and Left ventricular ejection fraction (LVEF) in a total of 16,923 European individuals with a maximum sample size of LVEDV (*n* = 16,920), LVESV (*n* = 16,920), LVEF (*n* = 16,923), and LV mass (*n* = 16,920) (18).

Estimates for CAD risk were obtained from the CARDIoGRAMplusC4D (CAD Genomewide Replication and Meta-analysis [CARDIOGRAM] plus the CAD [C4D] Genetics) Consortium's 1,000 Genomes–based transethnic meta-analysis of 60,801 case subjects and 123,504 control subjects (19). The majority (77%) of the participants were of European ancestry; 13% and 6% were of South Asian (India and Pakistan) and East Asian (China and Korea) ancestry, respectively, with smaller samples of Hispanic and African Americans. Case status was defined by an inclusive CAD diagnosis (for example, myocardial infarction, acute coronary syndrome, chronic stable angina, or coronary stenosis of >50%).

### 2.3. Selection of genetic instruments

**Figure 1:**
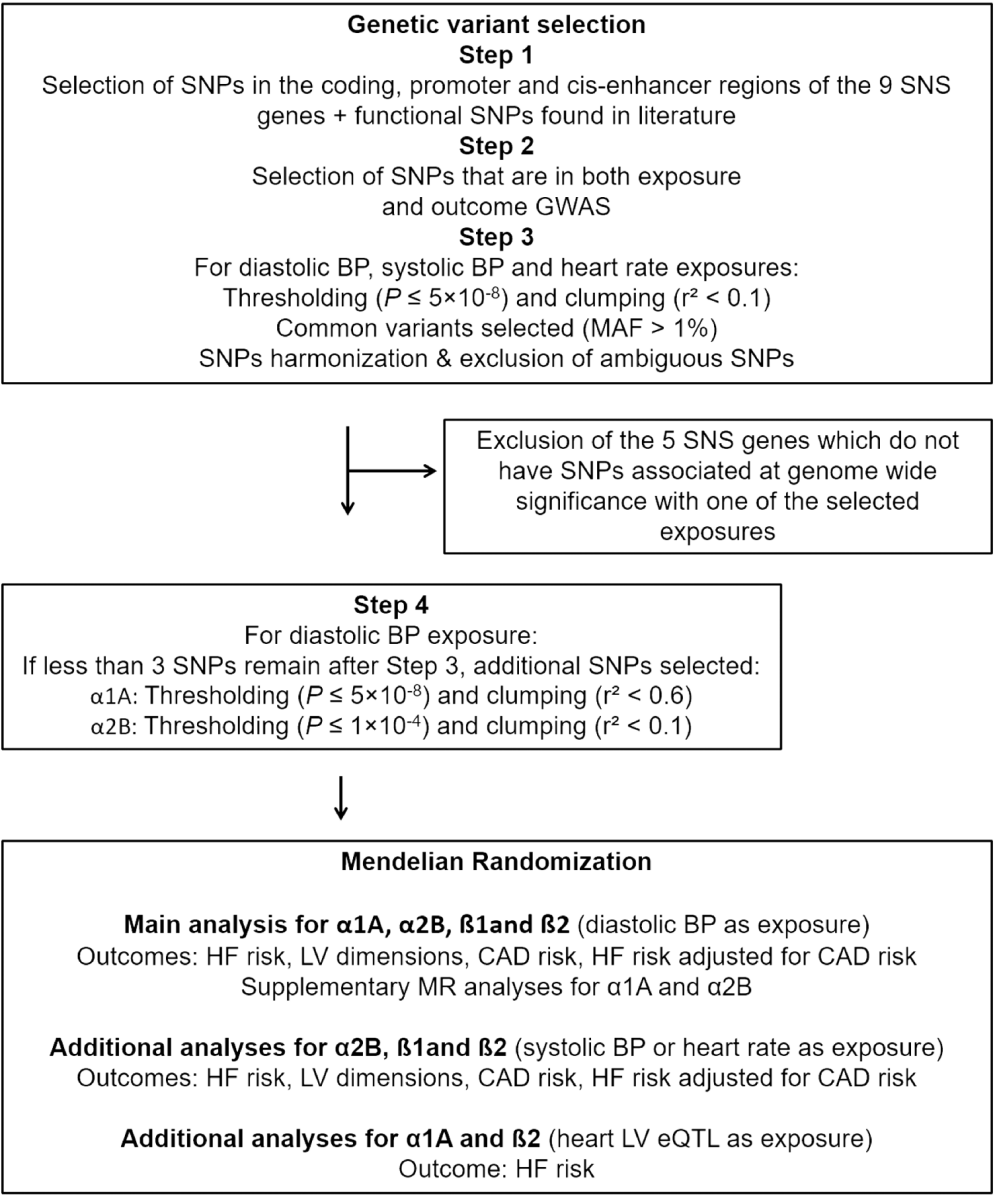
Design of the MR study for each of the nine-sympathetic nervous system (SNS) genes. Exposure GWAS obtained from: Blood pressure (BP), resting heart rate GWAS, and heart left ventricle (LV) expression quantitative trait loci (eQTL). Outcome GWAS obtained from: Heart failure (HF) risk (HERMES) GWAS after exclusion of the UK Biobank participants, GWAS of cardiac magnetic resonance imaging derived left ventricular (LV) dimensions, coronary artery disease (CAD) risk GWAS, and HERMES GWAS adjusted for CAD. GWAS, genome wide association studies; MR, mendelian randomization.

To build the genetic instrument, the gene encoding regions of the nine adrenergic receptors, ADRA1A (α1A), ADRA1B (α1B), ADRA1D (α1D), ADRA2A (α2A), ADRA2B (α2B), ADRA2C (α2C), ADRB1 (ß1), ADRB2 (ß2) and ADRB3 (ß3), as well as their promoter and cis-enhancer regions were first selected (Figure 1 and Supplementary Figure S2). This tailored approach, as contrary to the use of a fixed region upstream and downstream (+/− × kbp) of a gene, minimizes the risk of including non-relevant genetic variants that could bias the MR analysis. The cis-coding regions of the genes encoding the nine adrenergic receptors were defined using the Ensembl database (20). Promoter and cis-enhancer regions were identified using the GeneHancer database in the GeneCards online platform (version 4.8) (21).

We verified that the identified SNPs were specific to the genes encoding the adrenergic receptors when located in intergenic regions by using the genetic.opentargets.org database where we checked that the SNPs had a high Variants to Genes (V2G) score affiliated to the gene encoding the corresponding adrenergic receptor. The V2G score is a single aggregated score for variant-gene prediction obtained by combining eQTLs and pQTLs, chromatin interaction and conformation datasets, *in silico* functional predictions, and distance from the canonical transcript start site (22).

We then identified the genetic variants of these regions as single nucleotide polymorphisms (SNPs) that are in both exposure (BP, heart rate, LV eQTLs or blood pQTLs) and outcome GWAS and checked their specific relationship to the corresponding adrenergic receptor.

SNPs were then selected based on association with diastolic BP, systolic BP, or heart rate at genome-wide significance (*P* ≤ 5 × 10^–8^), with a Minor Allele Frequency (MAF) >0.01, and clumped to a linkage disequilibrium (LD) threshold of *r*^2^ < 0.1 using the 1000G European reference panel to ensure their independence.

When only one or two independent SNPs were found for a given gene using these criteria, we selected additional SNPs to perform MR analyses using the previously defined criteria, but with different thresholds either for their association with diastolic BP (*P* ≤ 1 × 10^−4^) or for LD clumping (*r*^2^ < 0.6).

The adrenoreceptor eQTLs were selected from the GTEx data file that contain the eGene and significant variant-gene associations based on permutations in the heart LV tissue and clumped to a LD threshold of *r*^2^ < 0.1.

After SNP selection, data of their associations with the BP, heart rate or LV expression exposures, and with the risk of HF (or LV dimensions/CAD outcomes) were harmonized to match coded effect alleles consistently.

Indeed, for each SNP we need to ensure that the measured effects on the exposure and the outcome correspond to the same effect alleles. It's worth noting that discrepancies might arise when comparing SNPs across different GWAS, necessitating a harmonization process before any subsequent analyses can be conducted. The methodology for SNP harmonization closely follows the approach outlined in the work by Hemani et al. (23).

To enhance the quality of our harmonization process, certain SNPs are excluded from consideration. Specifically, we exclude palindromic SNPs, which are characterized by having the same possible alleles on both the forward and reverse strands. Additionally, SNPs with major allele frequencies (MAF) approximating 50% (MAF > 0.42) are also omitted. This exclusion criteria is implemented to mitigate potential ambiguities in the subsequent analyses (24, 25).

We also searched for additional published SNPs, in particular for ADRA1A and ADRA2B (26–34) to ensure no SNP was missing. Finally, we looked for the linkage disequilibrium (LD) coefficients *r*^2^ between the SNPs described in the literature and the selected SNPs. In the end, we did not find any supplementary independent genetic variants that were significantly associated with the diastolic BP, systolic BP, or HR exposures.

#### 2.3.1. Genetic variants characterization

The genetic variants selected for the MR analysis were characterized with regard to their metabolic profile using the type 2 diabetes Knowledge Portal that enables the analysis of 325 cardio-metabolic traits in 281 datasets. A *p* = 0.05 significance threshold was used to examine the association between a genetic variant and a phenotype.

### 2.4. Statistical analysis

A two-sample MR study design with an approach relevant to drug target validation was used (9, 35).

When a single variant was available, the MR analyses were conducted using the Wald estimator, which is a causal estimate obtained for a single genetic variant by dividing its gene-outcome association by its gene-exposure association. For multiple variants, the fixed-effect “Inverse Variance Weighted” (IVW) method for correlated variants was employed.

This approach combines Wald ratio together in fixed effect meta-analysis, where the weight of each ratio is the inverse of the variance of the SNP-outcome association. Each instrumenting SNP is treated as an independent “study”, and the Wald ratios estimated for each SNP are meta-analysed under a fixed effects model. The fixed-effect “IVW” method for correlated variant is a specific parametrization of the generalized least squares technique that accounts for pairwise LD between variants at each locus using the 1000G European reference panel (36). Since the clumping threshold is not too strict (*R*^2^ > 0.1 or above) and variants from the same genetic region are used, they tend to still be in LD. It is therefore necessary to take in account their correlation. Furthermore, the adoption of a fixed-effects model assumes that all genetic variants are targeting the same causal effect parameter. Such assumption is reasonable when all the genetic variants are in the same gene region and then are likely to affect the risk factor in the same way.

Odds ratios (OR) were derived from the corresponding MR estimate for each adrenergic receptor and are given for a 1 mmHg decrease in BP or 1 beat per min (bpm) decrease in heart rate. For the LV dimensions, the MR results are expressed as effect size (β) and are given for a 1 mmHg decrease for BP or a 1 bpm decrease for heart rate, with a unit that depends on the LV dimension considered. For the MR analyses using eQTLs, the results are expressed as effect size weighted by the expression level of the gene encoding the corresponding adrenergic receptor.

Sensitivity analyses were employed to assess the validity of these findings. They included diagnostic tests for horizontal pleiotropy (Cochran's Q statistic and MREgger test) and SNP outliers (leave-one-out analysis). If horizontal pleiotropy was detected, then the MR Egger method would have been employed to conduct the MR analysis. When, for a given gene, additional SNPs have been selected using alternate thresholds, supplementary MR analyses using only the SNPs at genome-wide significance (*P* ≤ 5 × 10^–8^) and with a LD threshold of *r*^2 ^< 0.1 were also performed.

All analyses were conducted using the R programming language. The data formatting steps to perform MR analyses, including SNP selection and data harmonization steps, were completed using the R packages “data.table”, “sqldf”, “TwoSampleMR” (version 0.5.4) (23). MR estimates calculation, as well as sensitivity analyses, were performed using the R package MendelianRandomization (version 0.4.3) (37). GraphPad Prism software (version 7.03) was used to graphically display the MR analysis results.

### 2.5. Impact of CAD risk on HF risk by mediation analysis

For targets having a causal relationship with HF, we performed a mediation analysis to determine whether their impact on HF was mediated partially or not by an effect on the risk of CAD.

First, we performed MR analyses using CAD risk GWAS as outcome and the SNPs selected for the different exposure GWAS (BP and heart rate) to check whether these adrenergic receptors also influenced CAD risk. Then, to test whether the predicted effect on HF risk was mediated by the effect on CAD risk, we performed MR analyses using the previously obtained HF risk GWAS adjusted for CAD risk using Multi-trait Conditional and Joint Analysis (mtCOJO) as outcome (17). The mtCOJO method was used to check whether the target effect on HF changed after accounting for CAD, where a limited difference between the MR HF with and without CAD adjustment is indicative of an absence of mediation.

## 3. Results

The overall design and flow of the study are displayed in Figure 1.

### 3.1. Genetic variant selection

Among the nine sympathetic nervous system receptors, at least one SNP associated at genome-wide significance with BP or heart rate was identified in the cis region of genes encoding α1A (diastolic BP), α2B (diastolic BP and heart rate), ß1 and ß2 (diastolic and systolic BP) (Supplementary Figure S3, which corresponds to Figure 1 as well as Supplementary Tables S2, S3). While, several variants associated to BP or heart rate have been identified for the remaining genes, none of them have reached genome-wide significance.

All selected SNPs were used in all further MR analyses except those using LV dimensions as outcomes, as fewer α1A, ß1 and ß2 SNPs were in common between the BP and LV dimensions GWAS (Supplementary Table S3 and Figure S3).

### 3.2. Main analysis

The main analysis was performed with the genetic instruments weighted by diastolic BP as proxy for target activity since it was not possible to identify variants significantly associated with systolic BP and/or heart rate across all 4 genes. The OR derived from the corresponding MR estimate for each adrenergic receptor are given for a 1 mmHg decrease in BP.

#### 3.2.1. Heart failure MR

**Figure 2:**
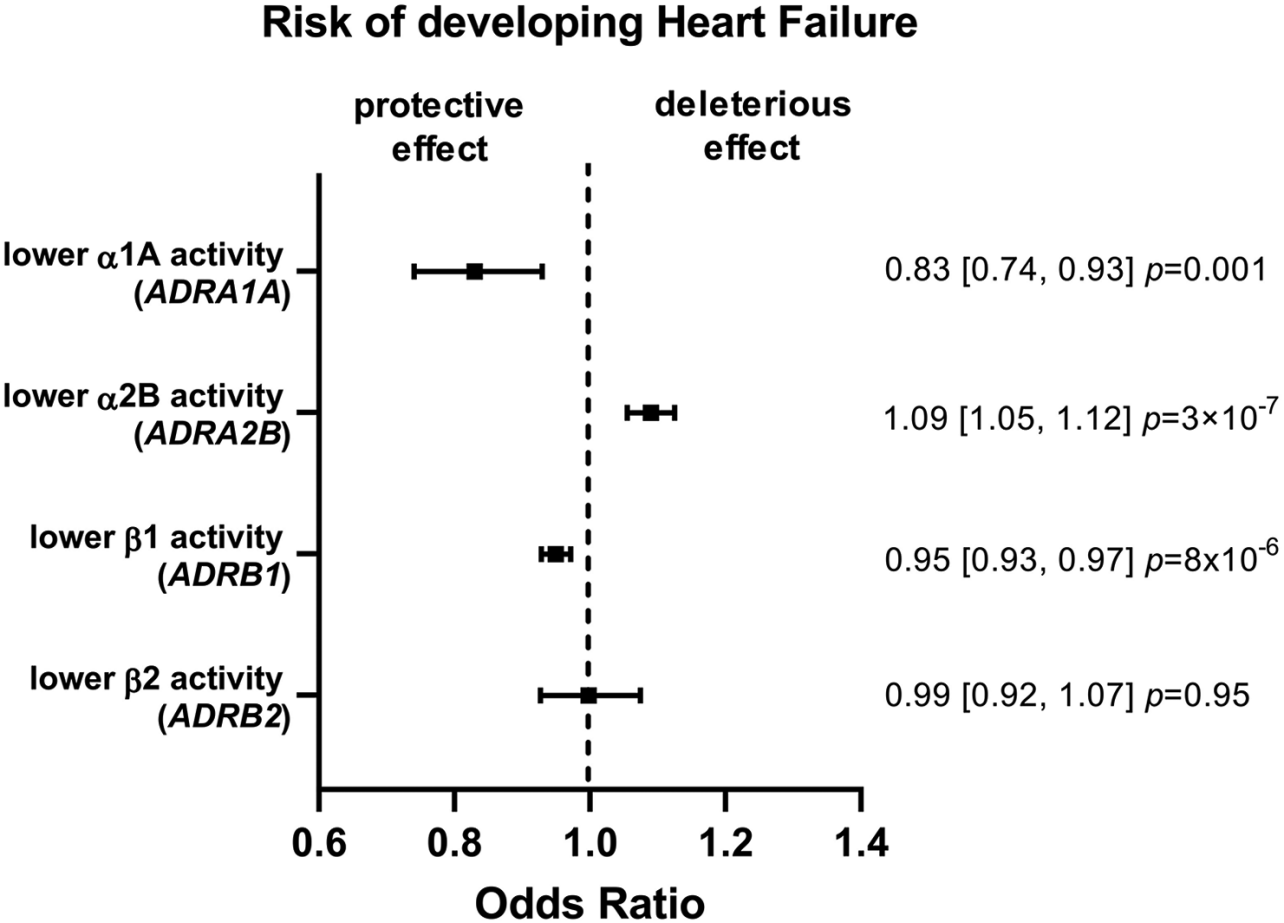
Mendelian randomization (MR) estimates showing the effect of the four adrenergic receptors activity on heart failure (HF) risk. The selected genetic instruments weighted by diastolic blood pressure (BP) (1 mmHg decrease) were used as proxy for the activity of each receptor. A lower α1A or ß1 activity was associated with a lower risk of developing HF, while an inverse relationship was found for a lower α2B activity and no evidence was found for an effect of ß2 activity.

A lower α1A or ß1 activity was associated with a lower risk of developing HF: OR 0.83 (95% CI 0.74–0.93, *P *= 0.001) and 0.95 (95% CI 0.93–0.97, *P *= 8 × 10^−6^) respectively (Figure 2 and Supplementary Figure S4). An inverse relationship was found for a lower α2B activity: OR 1.09 (95% CI 1.05–1.12, *P *= 3 × 10^−7^). No evidence was found for an effect of ß2 activity in HF risk: OR 0.99 (95% CI 0.92–1.07, *P *= 0.95).

#### 3.2.2. LV dimensions

**Figure 3:**
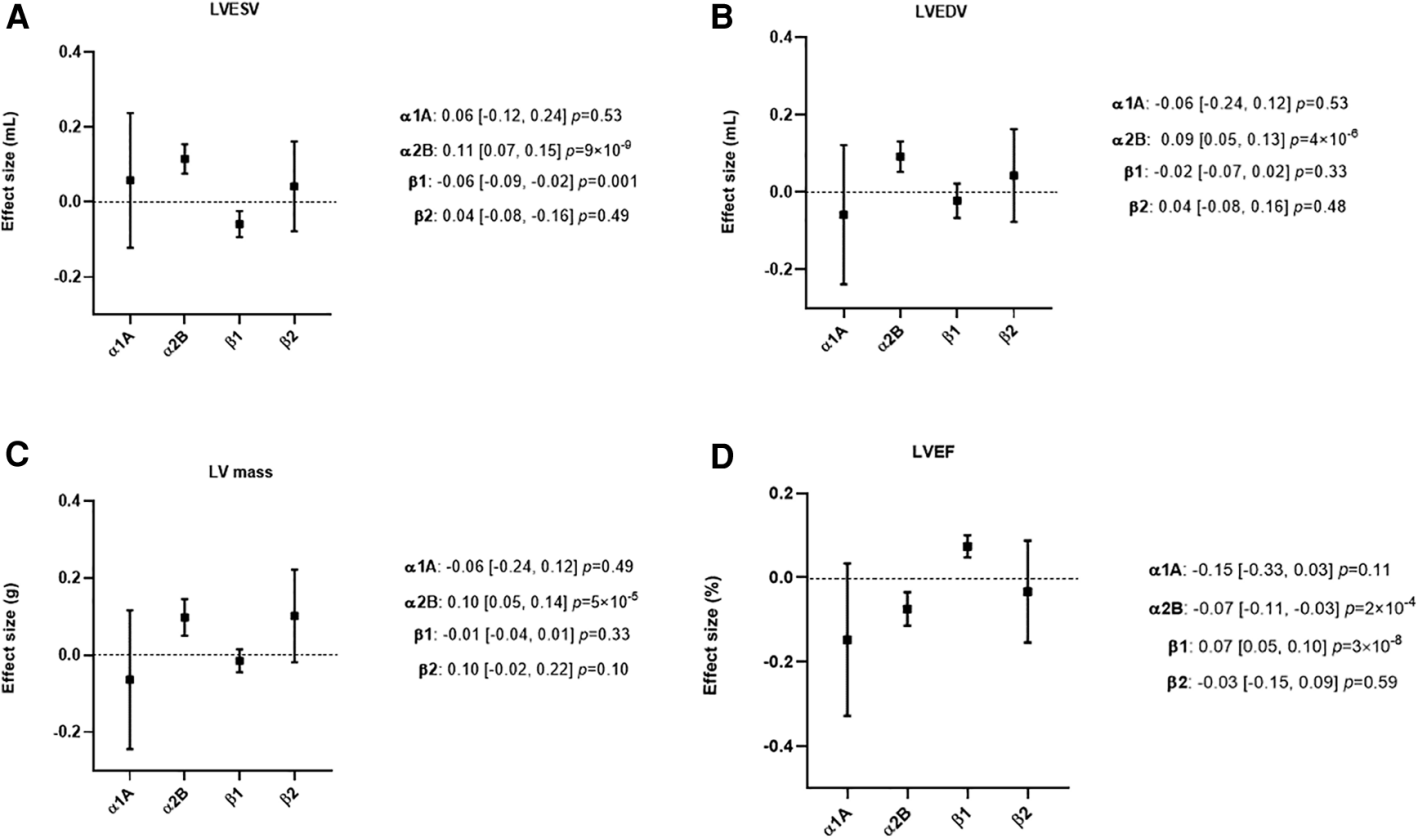
Mendelian randomization (MR) estimates showing the effect of the four adrenergic receptors, activity on left ventricular (LV) dimensions. The selected genetic instruments weighted by diastolic Blood Pressure (BP) (1 mmHg decrease) were used as proxy for the activity of each adrenergic receptor. The effect size is reported in mL for LV end-systolic volume (LVESV) and LV end-diastolic volume (LVEDV), in g for LV mass and in percentage for LV ejection fraction (LVEF). (**A**) MR results using LVESV as outcome. (**B**) MR results using LVEDV as outcome. (**C**) MR results using LV mass as outcome. (**D**) MR results using LVEF as outcome. A lower ß1 activity was associated with a lower LVESV and a higher LVEF, while a lower α2B activity was associated with a higher LVESV, LVEDV, LV mass and a lower LVEF.

MR analyses found no evidence for an effect of α1A or ß2 activity modulation on either LV volumes, LV mass, or LVEF (Figure 3, Supplementary Table S4).

A lower ß1 activity was associated with lower LVESV (*β* = −0.06 ml 95% CI −0.09 - −0.02, *P* = 0.001) and a higher LVEF (*β* = 0.07% 95% CI 0.05–0.10, *P *= 3 × 10^−8^). No evidence was found for an effect of ß1 on LVEDV or LV mass (Figure 3 and Supplementary Table S4).

A lower α2B activity was associated with higher LVESV (*β* = 0.11 ml 95% CI 0.07–0.15, *P *= 9 × 10^−9^), LVEDV (*β* = 0.09 ml 95% CI 0.05–0.13, *P *= 4 × 10^−6^), lower LVEF (*β* = −0.07% 95% CI −0.11–0.03, *P *= 2 × 10^−4^) and higher LV mass (*β* = 0.10 g 95% CI 0.05–0.14, *P *= 5 × 10^−5^) (Figure 3 and Supplementary Table S4).

#### 3.2.3. Coronary artery disease

**Figure 4:**
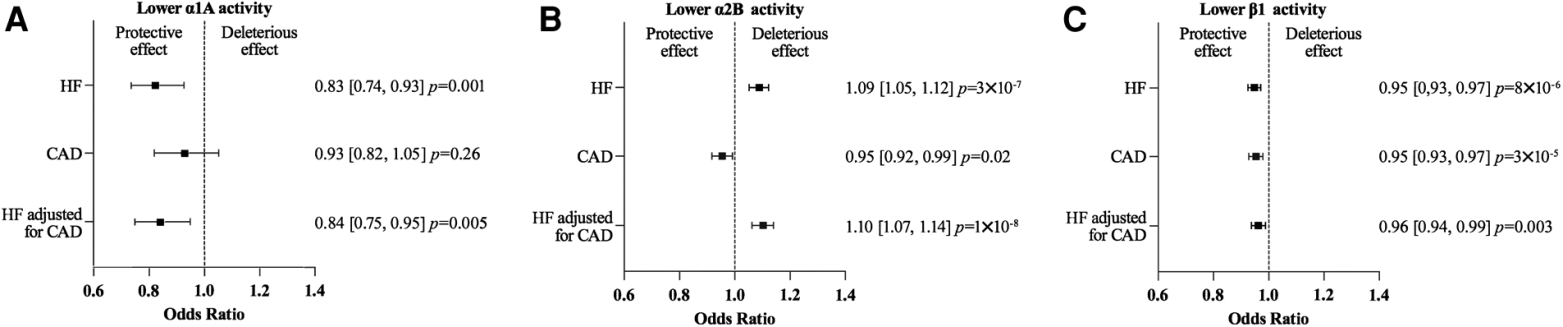
Mendelian randomization (MR) estimates showing the effect of α1A (**A**), α2B (**B**) and β1 (**C**) activity on heart failure (HF) risk, coronary artery disease (CAD) risk and HF risk adjusted for CAD risk. The selected genetic instruments weighted by diastolic Blood Pressure (1 mm Hg decrease) were used as proxy for the activity of each adrenergic receptor. No evidence was found for an effect of a lower α1A activity on the CAD risk. On the contrary a lower ß1 or α2B activity was associated with a lower CAD risk, but the magnitude of the effect of a lower α2B and ß1 activity activity on HF risk was similar using the while HF GWAS or the HF GWAS adjusted for CAD.

MR analyses found no evidence for an effect of α1A on CAD risk: OR 0.93 (95% CI 0.82–1.05, *P *= 0.26). A lower ß1 or α2B activity was associated with a lower CAD risk: OR 0.95 (95% CI 0.93–0.97, *P *= 3 × 10^−5^), and OR 0.95 (95% CI 0.92–0.99, *P *= 0.02), respectively (Figure 4 and Supplementary Table S5).

#### 3.2.4. Mediation of HF risk by CAD

The effects of a lower α1A, α2B, or ß1 activity on HF risk were similar when calculated using either the HF GWAS adjusted for CAD or the whole HF GWAS (Figure 4 and Supplementary Table S6).

### 3.3. Sensitivity and supplementary MR analyses

The sensitivity analyses had no substantive impact on the results presented above. In particular Cochran's Q statistic and MR-Egger tests were not significant, which rules out the presence of pleiotropy and the need to use MR Egger method. The supplementary MR analyses specific to α1A and α2B also agreed with the results presented above (Supplementary Figure S5).

### 3.4. Additional MR analyses

The analyses were repeated, but with the genetic instruments weighted by systolic BP as proxies for ß1 and ß2 activities and by heart rate as a proxy for α2B activity.

The results, as detailed in the supplemental Results, were consistent with the main analysis (Supplementary Tables S7–S9 and Figures S6–S9).

We repeated also these MR analyses using eQTL data which were available for ADRA1A and ABRB2. In general, these eQTL weighted analyses supported our findings, despite a decrease in precision related to the more limited number of available instruments (Supplementary Table S11).

We did not find any significant pQTLs for any of the adrenergic receptors studied, preventing any MR analyses with pQTLs.

## 4. Discussion

**Figure 5:**
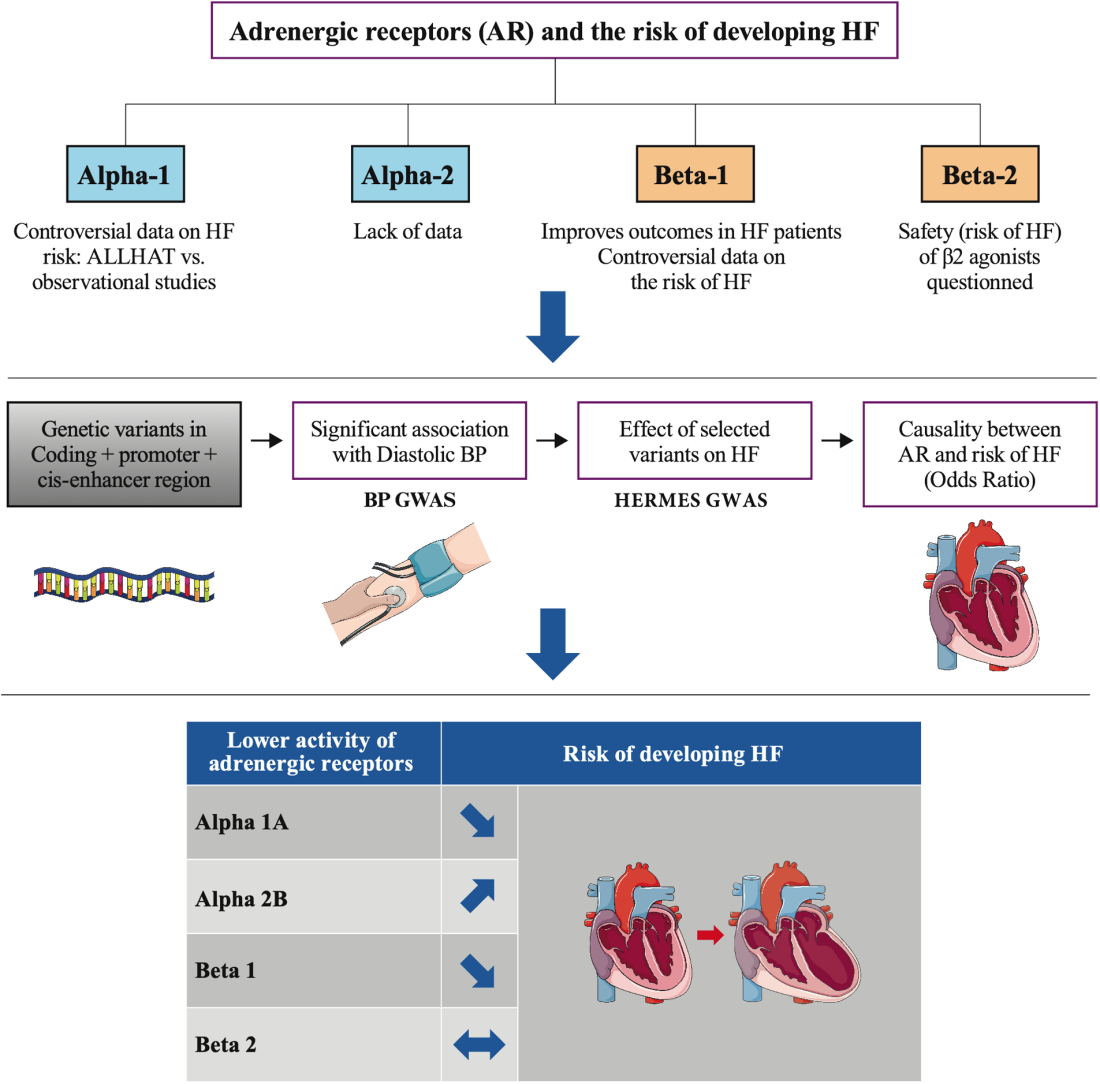
Lower genetic activity of 4 adrenoreceptors and the risk of heart failure.

Our study used drug target MR to recapitulate the effect of partial loss of function of several adrenoreceptors on the risk of developing HF and assess potential therapeutic actions of their modulation on this risk. It showed that genetically predicted lower ß1 or α1A activity is protective, whereas lower α2B activity is associated with higher HF risk. No evidence for a role of ß2 in HF was found (Figure 5).

Drug target MRs have recapitulated the results, positive or not, of randomized clinical trials performed in CAD (26, 38–40), but limited data on HF were available. Our findings confirmed their applicability in HF by re-demonstrating the known effect of ß1-blockade. By scaling the MR result to the average systolic BP-lowering effect of ß1 blockers (9.51 mmHg) (26) the OR for the risk of HF is 0.77 (95% CI 0.64–0.93, *P *= 0.008). Furthermore, our results are consistent with recent studies that suggest a lack of detrimental or beneficial effect of long-term ß2 modulation on LV volumes and function and the risk of HF (41).

Drug target MRs follow the same principle as the classical MR but they evaluate the effect of the drug target and not the biomarker itself on the disease (9). As the effect of variants on gene activity cannot usually be directly measured, a range of traits are used as proxies, including gene or protein expression, or downstream physiological biomarkers, such as in our study BP and heart rate, the main cardiovascular biomarkers modulated by the sympathetic nervous system. To be applicable, the method requires either a comprehensive understanding of the pathophysiology of the target such as in the case of our targets or reliable data on expression, available also in our study for ADRA1A and ADRB2.

Therefore, none of our analyses provide evidence that these drug target effects are mediated through BP or heart rate (9) as shown by the decrease in BP associated with a decrease in HF risk when secondary to a lower α1A or ß1 activity, or an increase when secondary to a lower α2B activity. Furthermore, MR estimates using variants modulating α1A or ß2 expression (eQTL) yield similar results as those modulating the function.

Adrenergic receptors modulate several mechanisms beyond BP and heart rate, including lipolysis or insulin secretion as shown by the association between several of our selected variants and the risk of diabetes and/or the lipid profile consistent with the known effect of the pharmacologic modulators (Supplementary Table S3). Such pleiotropic effects may participate in the relationships between the modulation of the various receptors we studied and the risk of HF.

We studied the effect of adrenoreceptors modulation on the risk of developing HF, but not in patients with HF, which would need additional studies. Our results suggest therefore that the benefits of ß1 blockers in HF extend to primary prevention. We found also, consistently with the results of clinical studies (7), that a lower genetic ß1 activity is associated with a decrease in CAD risk. Interestingly, our mediation analyses showed that this was not the cause of the protective role of lower ß1 activity on HF that was associated with an improvement in LV volume and function suggesting that this role may be secondary to the prevention of an adverse LV remodeling by blunting the sympathetic activity.

α1A adrenergic receptors are the most abundant alpha receptors in the heart and, contrary to β1-adrenergic receptors, are not downregulated in HF (42). There are contradictory data on the role of α1 blockers in HF. Doxazosin and prazosin were associated with an increased risk of HF in several studies, including the antihypertensive and lipid-lowering treatment to prevent heart attack trial (ALLHAT) and another trial comparing doxazosin to chlorthalidone (3, 5, 42). However, no direct comparison between α1 blockers and placebo in a large trial is available. On the contrary, non-specific α1 blockers were recently associated with an improvement in death and rehospitalization for HF, and specific α1A blockers with a neutral effect in a large HF cohort (6).

Consistently with ALLHAT findings, we did not find any effect of a lower α1A activity on the risk of CAD (5) that could explain the benefit on HF risk we predicted. α1 like ß1 chronic stimulation may have long-term deleterious effects on the LV, explaining the protective effect of lower α1 activity on the risk of HF.

α2A and α2C adrenergic receptors are expressed mainly in the central nervous system and their role seems to be mainly mediated by the modulation of sympathetic tone, while α2B adrenergic receptors, found more frequently in vascular smooth muscle (43), have a vasopressor effect and counteract the central hypotensive effects of α2A stimulation (43). We found several *ADRA2B* independent variants associated with BP and/or heart rate as previously reported (44), suggesting a potential role for this target in cardiovascular hemodynamic regulation.

Our analyses suggest that a lower α2B activity is associated with an adverse LV remodeling and an increase in HF risk. No specific α2 modulator has ever been tested in an HF trial. However, studies of two human α2B receptor variants suggested that they might protect cardiac muscle against sympathetic/catecholaminergic overstimulation (45, 46). Furthermore, recent preclinical studies underlined the potential of these receptors to safeguard cardiac muscle under adrenergic surge by governing intracellular Ca2 + handling and contractility (47, 48), and therefore reduce the risk of HF. α2B receptor stimulation induces also platelet aggregation (49). This may explain the protective effect of lower activity on the risk of CAD, which was however insufficient to counteract the increase in HF.

### 4.1. Strengths and limitations

The proposed methodology leverages downstream physiological biomarkers as proxies to evaluate the effect of gene activity. As a direct link between the modulation of the gene activity mediated by the SNPs and their signaling could not be established, we took great care to minimize the likelihood of attributing the observed effect to another protein coding gene. This was done by carefully selecting the SNPs using Genecard to identify introns, promoters and enhancers, rather than relying on a fixed region around the gene. Additionally, we confirmed the association of the variants with the corresponding gene using the V2G score from opentargets, and verified that the selected variants' effects on pleiotropic outcomes (such as glycemia and lipids) were consistent with the anticipated effects of target modulation. Finally, we searched for previously reported associations between the variants and the target in the literature. We then ensured the validity of our genetic instrument.

The robustness of our findings was ensured by the consistency with the sensitivity analyses that included supplemental analyses which used when possible additional exposures (systolic BP for ß1 and ß2, heart rate for α2B) or variants modulating gene expression (α1A and ß2).

However, while drug-target MRs are powerful tools to assess the presence and direction of the effect of the modulation of a potential target on a disease, their results may not be directly translated to the clinic due to the differences between genetic and pharmacological perturbation of a target including drug pharmacokinetics and duration of the intervention (lifelong for genetic). Nevertheless, our study gives relevant insights on the potential beneficial or harmful effects of the modulation of the adrenergic receptors on HF risk.

### 4.2. Conclusion

This drug target MR suggests that the inhibition of several adrenoreceptors may be preventive (α1A, and ß1), neutral (ß2) or detrimental (α2B) on the risk of developing HF. Furthermore, drug target MR can be considered a useful tool to identify and validate candidate targets in HF. This will help focusing on the most promising strategies that can lead to patient benefit, accelerate drug development, and limit studies of potentially non-efficacious drugs.

## Data Availability

The original contributions presented in the study are included in the article/**Supplementary Material**, further inquiries can be directed to the corresponding author.
