# Supplementary material for: Unraveling the relationships between alpha- and beta-adrenergic modulation and the risk of heart failure
Source: Front Cardiovasc Med. 2023 Oct 18;10:1148931. doi: 10.3389/fcvm.2023.1148931 (PMC10619754; doi:10.3389/fcvm.2023.1148931)
Supplement: Supplementary file 1 [file Datasheet1.pdf]

Supplementary Material

Unraveling the relationships between alpha- and beta-adrenergic modulation and the risk of Heart Failure

Claire Baudier, Françoise Fougerousse, Folkert W Asselbergs, Mickael Guedj, Michel Komajda, Dipak Kotecha, R.Thomas Lumbers, Amand F Schmidt, Benoît Tyl *.

*** Correspondence:** Benoît Tyl: benoit.tyl@gmail.com

1 Supplementary Results

Building of the genetic tools, additional information

Several ADRB1 variants were also associated with a change in heart rate, but the associated P-values were exceeding the genome-wide significance ($P > 5\times10^{-8}$). It was therefore not possible to weigh these instruments by heart rate to use them as proxies for ADRB1 activity in MR analyses.

Most variants used in our analyses have either been previously reported in the literature or are in LD ($r^2 > 0.1$) with variants from the literature (Supplementary Table 3).

Characterization of the variants

To assess the effect of the variants on the metabolic profile, we considered the association of a decrease in the target gene activity and the risk of diabetes and/or the relevant biomarkers.

Several ADRA1A (α1A), ADRA2B (α2B), and ADRB2 (β2) variants were associated with a decrease in the risk of diabetes and/or HBA1c, while several ADRB1 (β1) variants were associated with an increase in the risk of diabetes (Supplementary Table 3)

One ADRA1A (α1A) and one ADRB1 (β1) variant were associated with a decrease in HDL cholesterol. Two ADRB1 (β1) variants were associated with an increase in LDL cholesterol, while an ADRB2 (β2) variant was associated with the reverse (increase in HDL and decrease in LDL). Finally, several ADRB1 (β1) variants were associated with an increase in triglycerides.

Supplementary MR analyses for α1A and α2B

As specified in the “Selection of genetic instruments” section of the main manuscript, since less than three SNPs weighted by diastolic BP could be selected for α1A and α2B, we identified two additional α1A SNPs using the same previous criteria but with an associated P-value $\leq 1\times10^{-4}$; and three additional α2B SNPs using the same previous criteria but with a clumping threshold $r^2 < 0.6$ to perform the MR analyses (Figure 1 and Supplementary Figure 1, Supplementary Table 3).

Supplementary MR analyses for α1A and α2B were performed with only the SNPs selected using the same criteria as for ß1 and ß2: association at genome-wide significance ($P \leq 5\times10^{-8}$) and clumping at a LD threshold of $r^2 < 0.1$ (one SNP for α1A and two SNPs for α2B).

They provided similar results for HF risk (Supplementary Figure 3), LV dimensions, CAD risk, and HF risk-adjusted for CAD risk as outcomes.

There were two exceptions, both associated with the lowering of α2B: its deleterious effect on LVEF while numerically and directionally similar was no more statistically significant (β = -0.07 % 95% CI -0.16– 0.01, P=0.10), as well as its protective effect on the CAD risk (OR 0.96 95% CI 0.88–1.04, *P*=0.28).

Additional MR analyses with systolic BP or heart rate as proxies

The analyses were repeated, but this time with the genetic instruments weighted by systolic BP as proxies for ß1 and ß2 activities and by heart rate as a proxy for α2B activity. The ORs derived from the corresponding MR estimate for each adrenergic receptor are given a 1 beat per min (bpm) decrease in heart rate.

Heart failure

The results were consistent with the main analysis.

A lower ß1 activity was associated with a reduction in HF risk: OR 0.97 (95% CI 0.95–0.99, *P*=0.008; Supplementary Figure 4 and Supplementary Table 7), while no evidence was found for an effect of ß2 activity on HF risk: OR 0.98 (95% CI 0.93–1.02, *P*=0.34; Supplementary Table 7). A lower α2B activity was associated also with an increased risk of developing HF: OR 1.14 (95% CI 1.01–1.29, *P*=0.03; Supplementary Figure 5).

LV dimensions

Similar results as the main ones were found for ß1 (Supplementary Figure 8 and Supplementary Table 8) and ß2 (Supplementary Figure 8 and Supplementary Table 8) activity modulation regarding LV volumes, LV mass, and LVEF as outcome.

While the size and direction of the effects of a lower α2B activity on LVESV, LVEDV, LV mass, or LVEF were similar to those found in the main analyses, they were not statistically significant (Supplementary Figure 9).

Coronary Artery Disease and mediation analysis

A lower ß1 activity was associated with lower CAD risk (Supplementary Figure 6, Supplementary Table 9). However, contrary to the main analyses a lower α2B activity was not associated with CAD risk (Supplementary Figure 5).

MR mediation analyses using the HF GWAS adjusted for CAD found no statistically significant association between a lower ß1 activity and the risk of HF: OR 0.99 (95% CI 0.97–1.01, *P*=0.19) (Supplementary Figure 6 and Supplementary Table 10). The deleterious effect associated with a lower α2B activity on HF risk was similar when calculated using the HF GWAS adjusted for CAD or the whole HF GWAS: OR 1.14 (95% CI 1.01–1.29, *P*=0.03) (Supplementary Figure 5).

2 Supplementary Figures and Tables

2.1 Supplementary Figures

Supplementary Figure 1: Principle of Mendelian Randomization (MR) as a tool for drug target validation. The classical MR approach relies on the selection of variants in the whole genome that are associated at genome wide significance with the exposure of interest. Drug target MR follows the same principle as the classical MR method but restricts the genetic variants selection to the region of the gene encoding the drug target of interest, here one of the sympathetic nervous system genes. For each of these genes, the corresponding genetic variants are expected to affect its activity and then the biomarker trait of interest: blood pressure (BP) or heart rate, in this study. These traits are used to weight genetic variants for the drug target genes since the adrenergic receptor activities cannot be directly measured. The MR estimates calculated using these variants predict whether there is an effect of the modifiable exposure, i.e., the drug target activity, on the outcome, HF risk, left ventricular (LV) dimensions, CAD risk or HF risk adjusted for CAD risk.

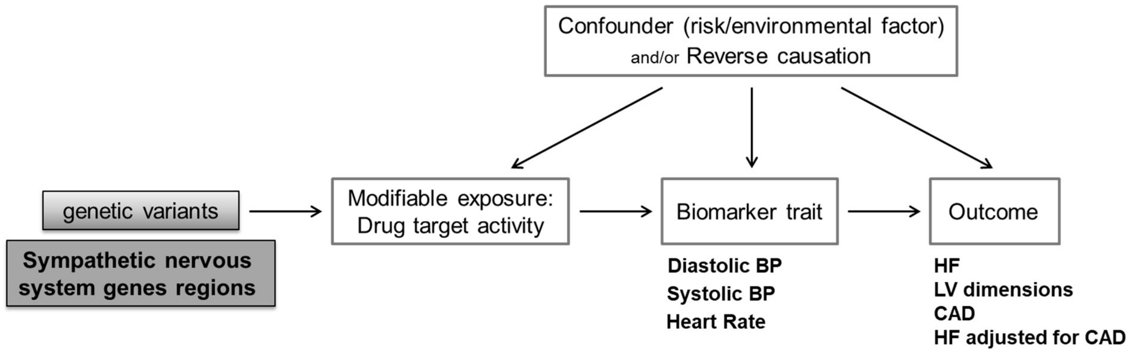

BP: Blood Pressure; CAD: Coronary Artery Disease; HF: Heart Failure; LV: Left Ventricular; MR: Mendelian Randomization.

Supplementary Figure 2: Scheme of the localization of the genetic variants used to build the genetic instrument. To build the genetic instrument, the gene encoding regions of the nine adrenergic receptors, ADRA1A (α1A), ADRA1B (α1B), ADRA1D (α1D), ADRA2A (α2A), ADRA2B (α2B), ADRA2C (α2C), ADRB1 (ß1), ADRB2 (ß2) and ADRB3 (ß3), as well as their promoter and cis-enhancer regions were first selected. As illustrated in this figure, the promoter and cis-enhancer regions were found either in the regions between the adrenergic receptor (AR) encoding region and the encoding region of the upstream or downstream genes, and/or within the encoding region of the upstream or downstream genes. The genetic variants used to build the genetic instrument were found within these regions. AR = adrenergic receptor.

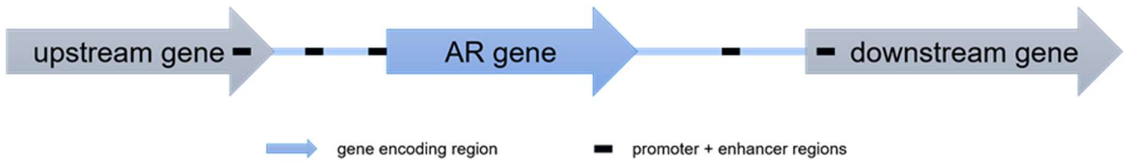

Supplementary Figure 3: Number of SNPs obtained after performing the different steps of the MR study for the genes (a) *ADRA1A* (α1A), (b) *ADRA2B* (α2B), (c) *ADRB1* (ß1) and (d) *ADRB2* (ß2). The steps 1, 2, 3, and 4 refer to the steps described in Figure 1.

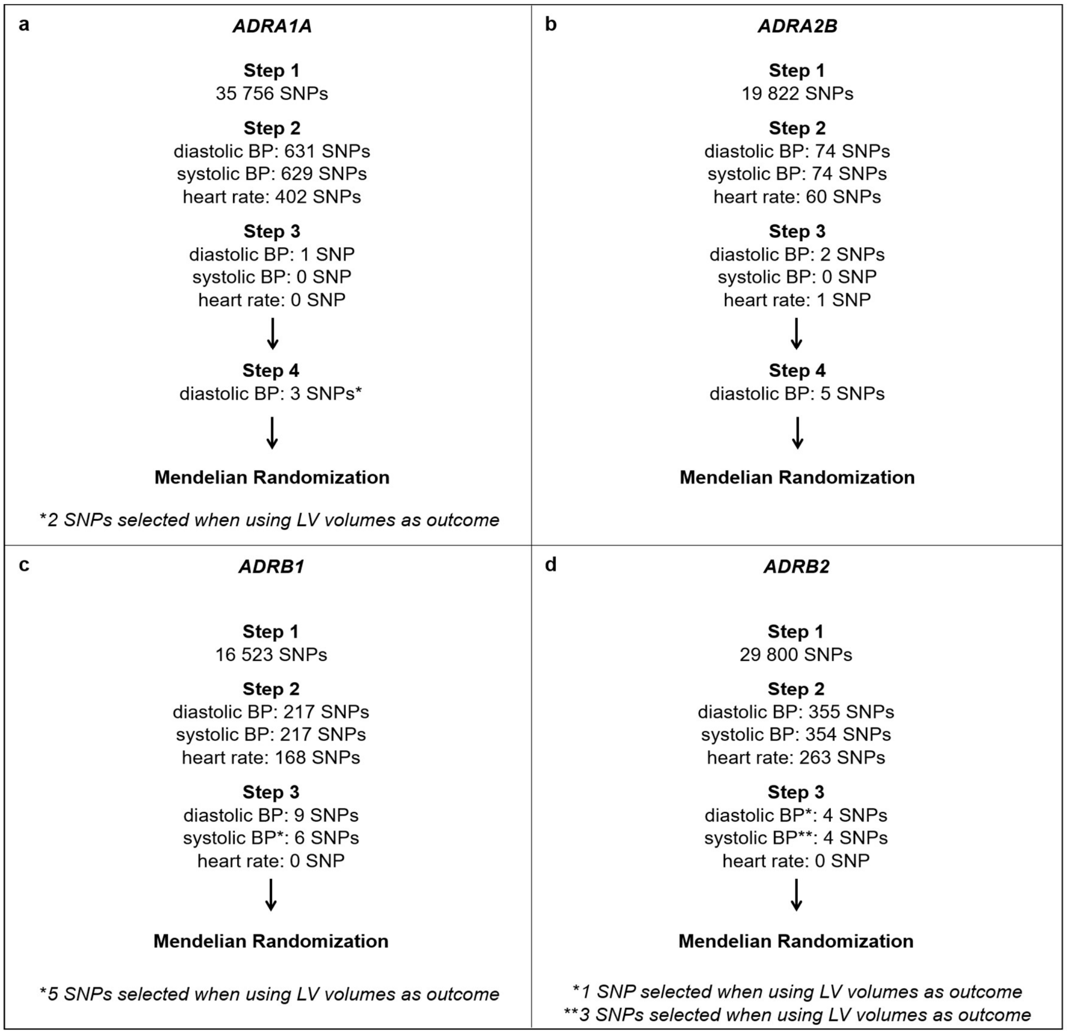

MR: Mendelian Randomization; SNP: Single Nucleotide Polymorphism

Supplementary Figure 4: Individual effect estimates of each SNP selected for the MR analyses showing the effect of α1A (a), β1 (b), α2B (c) and β2 (d) activity on heart failure risk. The selected genetic instruments weighted by **diastolic BP** (1 mm Hg decrease) were used as proxy for the activity of each adrenergic receptor. *BP: Blood Pressure; MR: Mendelian Randomization; SNP: Single Nucleotide Polymorphism.*

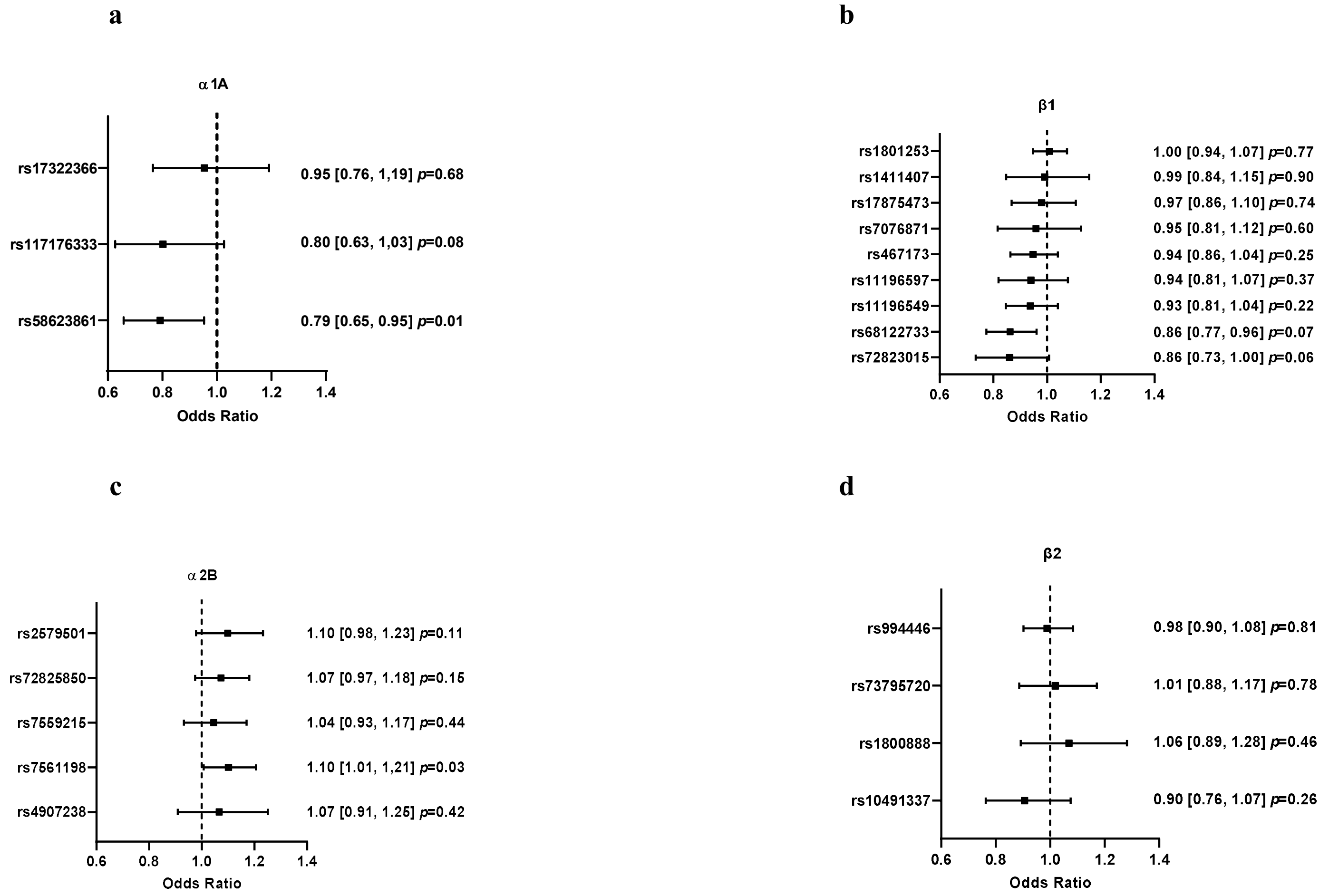

Supplementary Figure 5: Mendelian Randomization estimates showing the effect of ADRA1A and ADRA2B adrenergic receptors activity on Heart Failure risk using SNP selected with a threshold P value ≤ 5×10-8 and a threshold clumping $r^2 < 0.1$. The selected genetic instruments weighted by **diastolic Blood Pressure** (1 mm Hg decrease) were used as proxy for the activity of each receptor.

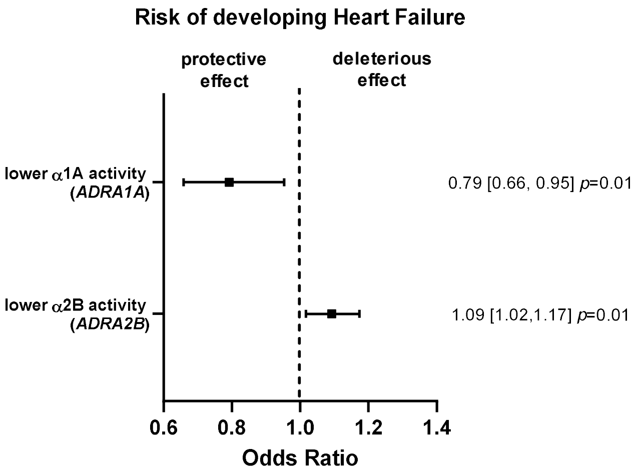

Supplementary Figure 6: MR estimates showing the effect of β1 activity on heart failure (HF) risk, coronary artery disease (CAD) risk and HF risk adjusted for CAD risk. The selected genetic instruments weighted by **systolic BP** (1 mm Hg decrease) were used as proxy for the activity of β1. *BP: Blood Pressure; CAD: Coronary Artery Disease; HF: Heart Failure; MR: Mendelian Randomization.*

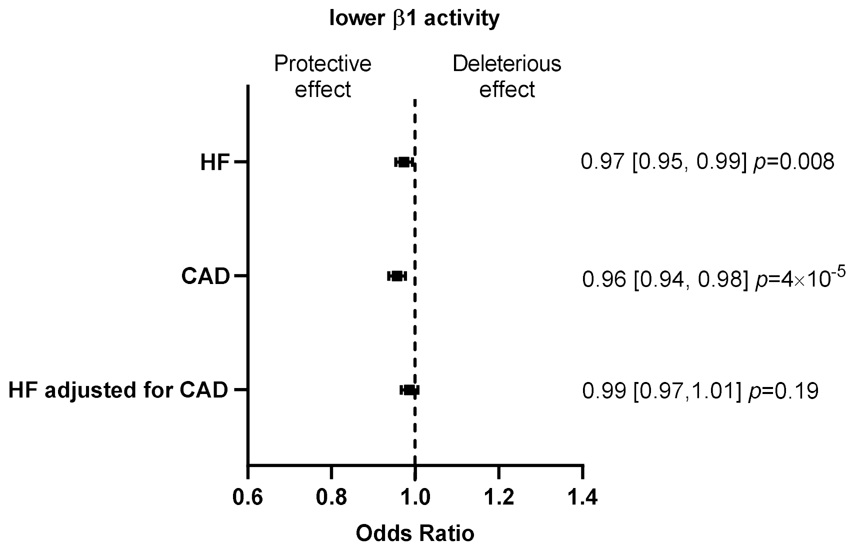

Supplementary Figure 7: MR estimates showing the effect of α2B activity on heart failure (HF) risk, coronary artery disease (CAD) risk and HF risk adjusted for CAD risk. The selected genetic instrument weighted by **heart rate** (1 beat per minute decrease) was used as proxy for the activity of α2B. The MR estimates were calculated with the Wald estimator method using the SNP rs749459.

CAD: Coronary Artery Disease; HF: Heart Failure; MR: Mendelian Randomization; SNP: Single Nucleotide Polymorphism.

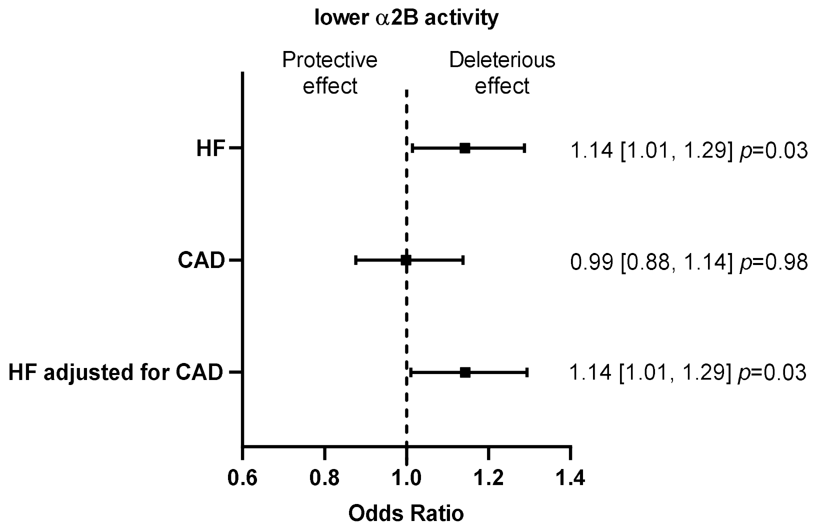

Supplementary Figure 8: MR estimates showing the effect of β1 and β2 activity on left ventricular dimensions (LVESV, LVEDV, LV mass and LVEF). The selected genetic instruments weighted by **systolic BP** (1 mm Hg decrease) were used as proxy for the activity of β1 and β2. The effect size is reported in mL for left ventricular end-systolic volume (LVESV) and left ventricular end-diastolic volume (LVEDV), in g for left ventricular (LV) mass and in percentage for left ventricular ejection fraction (LVEF). a: MR results using LVESV as outcome. b: MR results using LVEDV as outcome. c: MR results using LV mass as outcome. d: MR results using LVEF as outcome. *BP: Blood Pressure; MR: Mendelian Randomization.*

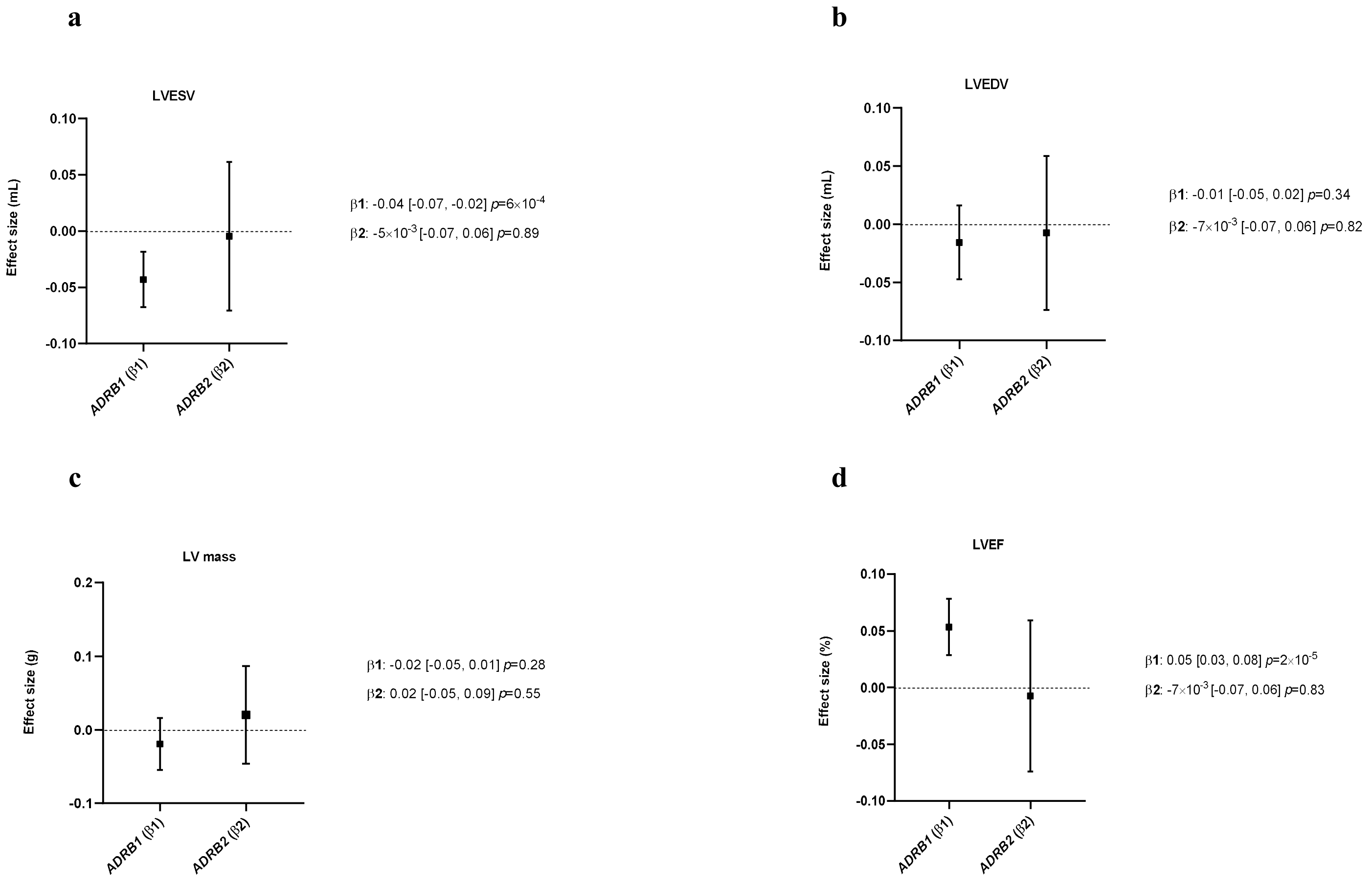

Supplementary Figure 9: MR estimates showing the effect of α2B activity on left ventricular dimensions (LVESV, LVEDV, LV mass and LVEF). The selected genetic instrument weighted by **heart rate** (1 beat per minute decrease) was used as proxy for the activity of α2B. The MR estimates were calculated with the Wald estimator method using the SNP rs749459. The effect size is reported in mL for left ventricular end-systolic volume (LVESV) and left ventricular end-diastolic volume (LVEDV), in g for left ventricular (LV) mass and in percentage for left ventricular ejection fraction (LVEF). a: MR result using LVESV as outcome. b: MR result using LVEDV as outcome. c: MR result using LV mass as outcome. d: MR result using LVEF as outcome. *MR: Mendelian Randomization; SNP: Single Nucleotide Polymorphism.*

a

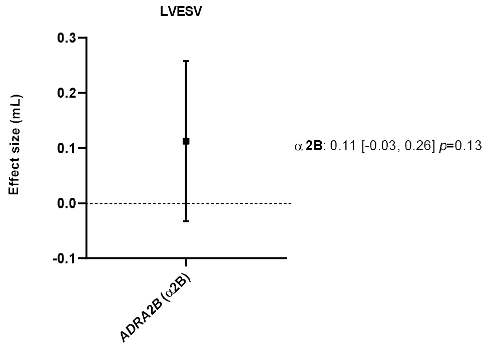

b

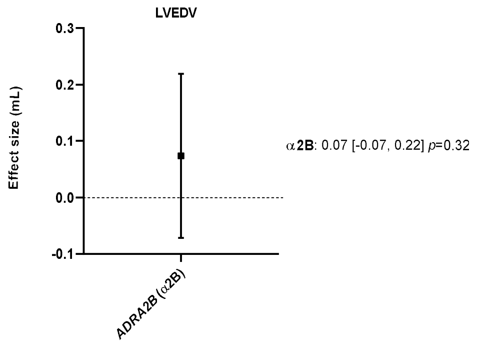

c

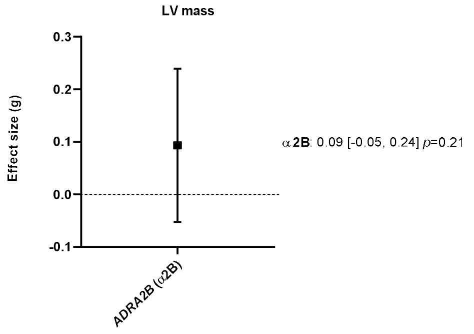

d

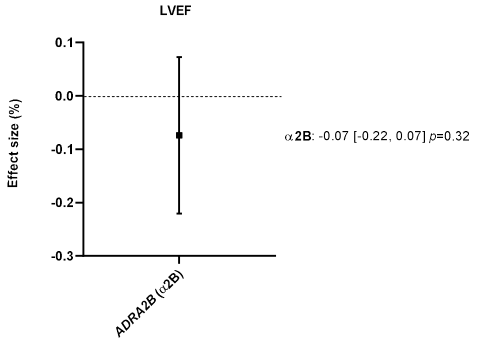

Supplementary Tables

Supplementary Table 1: Genome Wide Analysis Study (GWAS) summary statistics data source

Trait	Description	Cohort/ Consortium	Reference	Study-level association testing	Study	Data availability
Heart failure	a GWAS meta-analysis of 40,805 heart failure cases and 542,362 controls from 25 cohorts of European ancestry	HERMES except UK Biobank	1000G (Phase 1 and 3), HapMap2, HRC, population-specific reference	Logistic regression + cox proportional hazard (for incident cases)	Derived from Shah *et al.* (2020) [26]	Available upon reasonable request
Coronary artery disease	multi-ancestry GWAS meta-analysis of 60,801 CAD cases and 123,504 controls from 48 studies	CARDIoGR AMplusC4D	1000G Phase 1 v3	Logistic regression	Nikpay *et al.* (2015) [30]	https://www.ebi.ac.uk/gwas/studies/GCST003116
Heart failure adjusted for Coronary artery disease	a GWAS obtained by mtCOJO method using the heart failure and Coronary artery disease GWAS	HERMES and CARDIoGR AMplusC4D	1000G (Phase 1 and 3), HapMap2, HRC, population-specific reference	Multi-trait Conditional and Joint Analysis (mtCOJO)	Derived from Shah *et al.* (2020) [26] and Nikpay *et al.* (2015) [30]	Available upon reasonable request
LVESV, LVEDV, LV mass, LVEF	a GWAS of cardiac magnetic resonance imaging (MRI)-derived left ventricular measurements of 16923 European individuals from UK	UK Biobank	UK 10K, 1000 Genome Phase 3, HRC	Linear mixed-model with rank-based inverse- normal transformed phenotype	Aung *et al.* (2019) [29]	https://www.ebi.ac.uk/gwas/studies/GCST009397

	Biobank with a maximum sample size of LVEDV (n=16920), LVESV (n=16920), LVEF (n=16923), and LV mass (n=16920)					
Diastolic and systolic blood pressure	a GWAS meta-analysis of 757 601 individuals with European ancestry from the UK Biobank and the International Consortium of Blood Pressure (ICBP) GWAS meta-analysis	UK Biobank + International Consortium for Blood Pressure (ICBP)	1000 Genome Phase 1 v3, 1000G Phase 3, HRC, UK 10K	Linear mixed-model where association analysis was adjusted for sex, age, age², BMI<br><br>and fixed-effects inverse-variance-weighted used for meta-analysis	Evangelou E *et al.* (2018) [22]	https://www.ebi.ac.uk/gwas/studies/GCST006630 and https://www.ebi.ac.uk/gwas/studies/GCST006624
Resting heart rate	a GWAS of 458 969 individuals with European ancestry from UK Biobank	UK Biobank	UK 10K, 1000 Genome Phase 3	Linear mixed-model and association analysis was adjusted for age, sex, smoking, genotyping array, and 20 ancestry principal components.	Zhu Z *et al.* (2019) [23]	https://www.ebi.ac.uk/gwas/studies/GCST007609

LVEDV: Left ventricular end-diastolic volume; LVESV: Left ventricular end-systolic volume; LVEF: left ventricular ejection fraction; LV mass: Left ventricular mass.

The references number refer to the number of the manuscript in the supplemental references list

Supplementary Table 2: Sympathetic nervous system genes Single Nucleotide Polymorphisms (SNPs) associated at genome wide significance with at least one of the three exposures.

	GWAS		
	diastolic BP	systolic BP	heart rate
ADRA1A	**n=1**	n=0	n=0
ADRA2B	**n=2**	n=0	**n=1**
ADRB1	**n=9**	**n=6**	n=0
ADRB2	**n=4**	**n=4**	n=0

Number of SNPs reported for four sympathetic nervous system genes associated at genome wide significance (P value $\leq 5\times10^{-8}$) with at least one of the three biologic traits (diastolic BP, systolic BP and heart rate), with a Minor Allele Frequency (MAF) > 0.01 and clumped to a linkage disequilibrium (LD) threshold of $r^2 < 0.1$.

GWAS: Genome Wide Association Studies

Supplementary Table 3:

SNPs identified in the four adrenergic receptor genes *ADRA1A, ADRA2B, ADRB1* and *ADRB2* to perform MR analyses.

The SNPs reported in this table are the genetic variants that were used to perform the different MR analyses. Localization is reported for the corresponding gene indicated in the "gene" column. The Outcome GWAS column reports the MR analyses outcomes for which the SNPs were used: HF: heart failure, LVESV: left ventricular end-systolic volume, LVEDV: left ventricular end-diastolic volume, LV mass: left ventricular mass, LVEF: left ventricular ejection fraction, CAD: coronary artery disease, HFcondCAD: heart failure adjusted for coronary artery disease.

**Genes**	**Exposure**	**SNP**	**Chromosome Position**	**Localisation**	**EAF**	**EA/OA**	**Effect size**	**SE**	**P value**	**Outcome GWAS**	**Risk of Diabetes and/or HBA1c**	**Lipids Profile**	**Corresponding Literature [(1)]**
ADRA1A	diastolic BP	rs58623861	Chr8 26636981	Intron *ADRA1A*	0.2553	A/ G	0.1079	0.0199	5.64×10^{-8}	**HF**, LVESV, LVEDV, LV mass, LVEF, CAD, HFcondCAD	Decrease	HDL-c decrease	[38,40,41]
	diastolic BP	rs117176333*	Chr8 26611719	Intron *ADRA1A*	0.9677	A/ C	-0.2204	0.0502	1.11×10^{-5}	**HF**, CAD,HFcondCAD	Decrease		[41]
	diastolic BP	rs17322366*	Chr8 26486106	Enhancer	0.8245	T/ C	0.0992	0.0227	1.23×10^{-5}	**HF**, LVESV, LVEDV, LV mass, LVEF, CAD, HFcondCAD			
	LV eQTL	rs7833391	Chr8 26786959	intron	0.3614	G/C	0,1301	0,0275	$3,41\times10^{-6}$	**HF**		HDL-c decrease	[38,40,41]
ADRA2B	diastolic BP	rs4907238	Chr2 97197195	Promoter/ enhancer	0.7181	A/ G	0.12	0.0195	7.03×10^{-10}	**HF**, LVESV, LVEDV, LV mass, LVEF, CAD, HFcondCAD	Decrease		[36]

		rs7561198	Chr2 96774981	Enhancer	0.341	C/ G	0.2004	0.0183	5.39×10^{-28}	**HF**, LVESV, LVEDV, LV mass, LVEF, CAD, HFcondCAD			[36]
		rs2579501**	Chr2 97199251	Promoter/ enhancer	0.3395	A/ G	0.1563	0.0183	1.12×10^{-17}	**HF**, LVESV, LVEDV, LV mass, LVEF, CAD, HFcondCAD	Decrease		[36]
		rs72825850**	Chr2 96825851	Promoter/ enhancer	0.7828	A/ G	-0.2223	0.022	4.52×10^{-24}	**HF**, LVESV, LVEDV, LV mass, LVEF, CAD, HFcondCAD			[36]
		rs7559215**	Chr2 96874307	Promoter/ enhancer	0.8612	C/ G	-0.2345	0.0267	1.5×10-18	**HF**, LVESV, LVEDV, LV mass, LVEF, CAD, HFcondCAD			[36]
	heart rate	rs749459	Chr2 96795714	Enhancer	0.3716	G/ A	-0.1496	0.02317	2.6×10^{-11}	**HF**, LVESV, LVEDV, LV mass, LVEF, CAD, HFcondCAD			[36]
ADRB1	diastolic BP	rs11196549	Chr10 115707298	Enhancer	0.0426	A/ G	0.4454	0.0448	2.94×10^{-23}	**HF**, LVESV, LVEDV, LV mass, LVEF, CAD, HFcondCAD			[10]
		rs11196597	Chr10 115788094	Enhancer	0.1329	A/ G	0.1817	0.0262	3.82×10^{-12}	**HF**, CAD, HFcondCAD	Increase	Increase in TG	[10,43]
		rs1411407	Chr10 115747786	Enhancer	0.4757	T/ C	-0.1109	0.0176	3.35×10^{-10}	**HF**, LVESV, LVEDV, LV mass, LVEF, CAD, HFcondCAD		HDL-c decrease	
		rs17875473	Chr10 115800294	Enhancer	0.0865	T/ C	0.2459	0.0317	8.64×10^{-15}	**HF**, LVESV, LVEDV, LV mass, LVEF, CAD, HFcondCAD	Increase		[10]

		rs1801253	Chr10 115805056	Promoter/ enhancer missense	0.7345	C/ G	0.3183	0.0197	1.57×10^{-58}	**HF**, LVESV, LVEDV, LV mass, LVEF, CAD, HFcondCAD	Increase	Increase in TG	[10,37,41]
		rs467173	Chr10 115721430	Enhancer	0.3271	T/ G	-0.1985	0.0186	1.21×10^{-26}	**HF**, LVESV, LVEDV, LV mass, LVEF, CAD, HFcondCAD		Increase in TG	
		rs68122733	Chr10 115831533	Enhancer	0.8283	A/ G	0.2104	0.0232	1.34×10^{-19}	**HF**, LVESV, LVEDV, LV mass, LVEF, CAD, HFcondCAD			[10,37,41]
		rs7076871	Chr10 115842297	Enhancer	0.3517	T/ C	0.1117	0.0184	1.18×10^{-9}	**HF**, LVESV, LVEDV, LV mass, LVEF, CAD, HFcondCAD			[41]
		rs72823015	Chr10 115786611	Enhancer	0.1239	T/ G	0.1714	0.0265	9.34×10^{-11}	**HF**, LVESV, LVEDV, LV mass, LVEF, CAD, HFcondCAD		Increase in TG and LDL-c	
		rs717368	Chr10 115707296	Enhancer	0.9155	A/ G	-0.2035	0.0325	3.68×10^{-10}	LVESV, LVEDV, LV mass, LVEF			[10]
	systolic BP	rs11196549	Chr10 115707298	Enhancer	0.0425	A/ G	0.6884	0.0784	1.58×10^{-18}	**HF**, LVESV, LVEDV, LV mass, LVEF, CAD, HFcondCAD			[10]
		rs11196597	Chr10 115788094	Enhancer	0.133	A/ G	0.2858	0.0458	4.23×10^{-10}	**HF**, CAD, HFcondCAD			[10,43]

		rs17875473	Chr10 115800294	Enhancer	0.0871	T/ C	0.3283	0.0552	2.66×10^{-9}	**HF**, LVESV, LVEDV, LV mass, LVEF, CAD, HFcondCAD			10
		rs1801253	Chr10 115805056	Promoter/ enhancer missense	0.7338	C/ G	0.4626	0.0344	2.84×10^{-41}	**HF**, LVESV, LVEDV, LV mass, LVEF, CAD, HFcondCAD			10,37,41
		rs68122733	Chr10 115831533	Enhancer	0.8282	A/ G	0.3343	0.0406	1.69×10^{-16}	**HF**, LVESV, LVEDV, LV mass, LVEF, CAD, HFcondCAD			10,37,41
		rs460718	Chr10 115721364	Enhancer	0.3266	A/ G	-0.2764	0.0324	1.36×10^{-17}	**HF**, LVESV, LVEDV, LV mass, LVEF, CAD, HFcondCAD		Increase in TG and LDL-c	41
ADRB2	diastolic BP	rs10491337	Chr5 148331046	Enhancer	0.0468	T/ G	-0.2395	0.0429	2.28×10^{-8}	**HF**, CAD, HFcondCAD	Decrease		
		rs1800888	Chr5 148206885	*ADRB2* missense	0.0143	T/ C	0.4644	0.0764	1.22×10^{-9}	**HF**, CAD, HFcondCAD		Decrease in LDL, increase HDL	41
		rs73795720	Chr5 148331976	Enhancer	0.0447	T/ C	-0.3129	0.0428	2.52×10^{-13}	**HF**, CAD, HFcondCAD			
		rs994446	Chr5 148348395	Enhancer	0.2083	A/ G	-0.2212	0.0214	6.01×10^{-25}	**HF**, LVESV, LVEDV, LV mass, LVEF, CAD, HFcondCAD	Decrease		
	systolic BP	rs10491337	Chr5 148331046	Enhancer	0.0476	T/ G	-0.433	0.0742	5.27×10^{-9}	**HF**, CAD, HFcondCAD			

		rs994446	Chr5 148348395	Enhancer	0.2086	A/ G	-0.3466	0.0374	1.87×10^{-20}	**HF**, LVESV, LVEDV, LV mass, LVEF, CAD, HFcondCAD			
		rs17640858	Chr5 148346260	Enhancer	0.3687	A/ C	0.2014	0.0321	3.3×10^{-10}	**HF**, LVESV, LVEDV, LV mass, LVEF, CAD, HFcondCAD	Decrease		
		rs4579247	Chr5 148520213	Promoter/ enhancer	0.2438	T/ C	0.219	0.0356	7.45×10^{-10}	**HF**, LVESV, LVEDV, LV mass, LVEF, CAD, HFcondCAD	Decrease		
	LV eQTL	rs1036175	Chr5 148874294	regulatory region	0.3743	G/C	-0,1643	0,0424	$1,31\times10^{-4}$	**HF**			

Chr: chromosome, Pos: position (hg19), EA: effect allele, OA: other allele, EAF: effect allele frequency, SE: standard error of the effect size

SNP: single nucleotide polymorphisms; MR: Mendelian randomization; GWAS: Genome Wide Analysis Study; BP: Blood Pressure; LD: linkage disequilibrium. LV: Left Ventricle; eQTL: expression quantitative trait loci

* These SNPs were selected using a threshold P value $\leq 1\times10^{-4}$ and a threshold clumping $r^2 < 0.1$

** These SNPs were selected using a threshold P value $\leq 5\times10^{-8}$ and a threshold clumping $r^2 < 0.6$

(1) Corresponding literature: Literature either citing the SNP or associated SNP (LD $r^2 > 0.1$). The number refer to the number of the manuscript in the supplemental references list

Supplementary Table 4

Individual effect estimates of each SNP selected for the MR analyses showing the effect of α1A (*ADRA1A*), α2B (*ADRA2B*), β1 (*ADRB1*) and β2 (*ADRB2*) activity on left ventricular dimensions (LVESV, LVEDV, LV mass and LVEF). The selected genetic instruments weighted by **diastolic BP** (1 mm Hg decrease) were used as proxy for the activity of α1A, α2B, β1 and β2. The effect size is reported in mL for left ventricular end-systolic volume (LVESV) and left ventricular end-diastolic volume (LVEDV), in g for left ventricular (LV) mass and in percentage for left ventricular ejection fraction (LVEF).

SNP: single nucleotide polymorphisms; MR: Mendelian randomization; BP: Blood Pressure

Genes	End points (unit)	SNP	Effect size	Low 95% CI	Upp 95% CI	*P* value
ADRA1A	LVESV (mL)	rs58623861	0.11	-0.11	0.33	0.33
		rs17322366	-0.03	-0.31	0.25	0.85
		Fixed-effect IVW	0.06	-0.12	0.24	0.53
	LVEDV (mL)	rs58623861	-0.01	-0.24	0.21	0.90
		rs17322366	-0.13	-0.41	0.15	0.35
		Fixed-effect IVW	-0.06	-0.24	0.12	0.53
	LV mass (g)	rs58623861	0.05	-0.17	0.27	0.65
		rs17322366	-0.25	-0.53	0.03	0.08
		Fixed-effect IVW	-0.06	-0.24	0.12	0.49
	LVEF (%)	rs58623861	-0.15	-0.38	0.07	0.17
		rs17322366	-0.13	-0.42	0.15	0.34
		Fixed-effect IVW	-0.15	-0.33	0.03	0.11
ADRA2B	LVESV (mL)	rs7561198	0.12	0.008	0.23	0.03

		rs4907238	0.17	-0.02	0.37	0.08
		rs2579501	0.08	-0.06	0.22	0.28
		rs7559215	0.09	-0.04	0.23	0.17
		rs72825850	0.11	-0.004	0.23	0.06
		Fixed-effect IVW	0.11	0.07	0.15	9×10-9
	LVEDV (mL)	rs7561198	0.08	-0.03	0.20	0.14
		rs4907238	0.20	0.002	0.39	0.05
		rs2579501	0.06	-0.08	0.20	0.39
		rs7559215	0.10	-0.03	0.24	0.13
		rs72825850	0.10	-0.02	0.21	0.10
		Fixed-effect IVW	0.09	0.05	0.13	4×10^{-6}
	LV mass (g)	rs7561198	0.10	-0.01	0.21	0.09
		rs4907238	0.31	0.11	0.51	0.002
		rs2579501	0.09	-0.05	0.23	0.21
		rs7559215	0.12	-0.01	0.26	0.07
		rs72825850	0.09	-0.03	0.20	0.13
		Fixed-effect IVW	0.10	0.05	0.14	5×10^{-5}
	LVEF (%)	rs7561198	-0.09	-0.21	0.02	0.10
		rs4907238	-0.03	-0.22	0.17	0.79
		rs2579501	-0.05	-0.20	0.09	0.44
		rs7559215	-0.04	-0.17	0.09	0.56

		rs72825850	-0.06	-0.18	0.06	0.31
		Fixed-effect IVW	-0.07	-0.11	-0.03	2×10^{-4}
ADRB1	LVESV (mL)	rs717368	-0.17	-0.35	0.02	0.08
		rs467173	-0.11	-0.22	0.005	0.06
		rs11196597	-0.11	-0.28	0.06	0.22
		rs7076871	-0.10	-0.29	0.10	0.34
		rs1801253	-0.07	-0.15	0.002	0.06
		rs68122733	-0.006	-0.14	0.13	0.92
		rs17875473	0.008	-0.15	0.16	0.92
		rs72823015	0.13	-0.05	0.32	0.16
		rs1411407	0.14	-0.05	0.34	0.15
		Fixed-effect IVW	-0.06	-0.09	-0.02	0.001
	LVEDV (mL)	rs717368	-0.20	-0.39	-0.02	0.03
		rs467173	-0.11	-0.22	0.005	0.06
		rs11196597	-0.09	-0.26	0.08	0.29
		rs7076871	-0.05	-0.24	0.15	0.65
		rs1801253	-0.02	-0.09	0.06	0.61
		rs68122733	0.03	-0.10	0.16	0.67
		rs17875473	0.08	-0.07	0.23	0.31
		rs72823015	0.16	-0.03	0.34	0.09
		rs1411407	0.18	-0.02	0.37	0.07
		Fixed-effect IVW	-0.02	-0.07	0.02	0.33

	LV mass (g)	rs467173	-0.11	-0.22	0.009	0.07
		rs1801253	-0.06	-0.14	0.01	0.11
		rs1411407	-0.05	-0.24	0.15	0.63
		rs17875473	-0.03	-0.18	0.13	0.72
		rs11196597	-0.004	-0.18	0.17	0.96
		rs72823015	0.001	-0.18	0.19	0.99
		rs717368	0.004	-0.18	0.19	0.96
		rs7076871	0.04	-0.16	0.24	0.68
		rs68122733	0.11	-0.02	0.24	0.11
		Fixed-effect IVW	-0.01	-0.04	0.01	0.33
	LVEF (%)	rs1411407	-0.04	-0.23	0.16	0.70
		rs72823015	0.01	-0.17	0.20	0.87
		rs717368	0.02	-0.16	0.21	0.82
		rs467173	0.05	-0.07	0.16	0.42
		rs17875473	0.05	-0.10	0.21	0.48
		rs11196597	0.06	-0.11	0.24	0.46
		rs68122733	0.07	-0.07	0.20	0.32
		rs7076871	0.08	-0.11	0.28	0.41
		rs1801253	0.10	0.02	0.18	0.01
		Fixed-effect IVW	0.07	0.05	0.10	3×10^{-8}
ADRB2	LVESV (mL)	rs994446	0.04	-0,08	0.16	0.50

	LVEDV (mL)		0.04	-0.08	0.16	0.48
	LV mass (g)		0.10	-0.02	0.22	0.10
	LVEF (%)		-0.03	-0.15	0.09	0.59

SNP: single nucleotide polymorphisms; MR: Mendelian randomization; BP: Blood Pressure

Supplementary Table 5

Individual effect estimates of each SNP selected for the MR analyses showing the effect of α1A (*ADRA1A),* α2B (*ADRA2B*) and β1 (*ADRB1*) activity on coronary artery disease risk. The selected genetic instruments weighted by **diastolic BP** (1 mm Hg decrease) were used as proxy for the activity of α1A, α2B and β1.

Genes	SNPs	Odds ratio	Low 95% CI	Upp 95% CI	P value
ADRA1A	rs58623861	1.02	0.84	1.24	0.84
	rs117176333	0.88	0.66	1.16	0.36
	rs17322366	0.80	0.63	1.03	0.08
	Fixed-effect IVW	0.93	0.82	1.05	0.26
ADRA2B	rs4907238	0.92	0.78	1.10	0.38
	rs7559215	0.87	0.76	1.01	0.07
	rs72825850	0.94	0.83	1.06	0.32
	rs2579501	0.95	0.84	1.09	0.48
	rs7561198	0.97	0.88	1.07	0.56
	Fixed-effect IVW	0.95	0.92	0.99	0.02
ADRB1	rs1411407	0.86	0.73	1.01	0.07
	rs11196549	0.86	0.77	0.97	0.01
	rs17875473	0.87	0.74	1.01	0.07
	rs467173	0.93	0.85	1.03	0.17
	rs68122733	0.94	0.83	1.06	0.29
	rs1801253	0.95	0.89	1.02	0.17

	rs11196597	0.96	0.84	1.09	0.52
	rs72823015	0.97	0.81	1.17	0.74
	rs7076871	1.04	0.87	1.23	0.68
	Fixed-effect IVW	0.95	0.93	0.97	3×10^{-5}

Supplementary Table 6

Individual effect estimates of each SNP selected for the MR analyses showing the effect of of α1A (*ADRA1A*), α2B (*ADRA2B*) and β1 (*ADRB1*) activity on heart failure risk adjusted for coronary artery disease risk. The selected genetic instruments weighted by **diastolic BP** (1 mm Hg decrease) were used as proxy for the activity of α1A, α2B, and β1.

Genes	SNPs	Odds ratio	Low 95% CI	Upp 95% CI	*P* value
ADRA1A	rs58623861	0.79	0.65	0.95	0.01
	rs117176333	0.83	0.64	1.07	0.16
	rs17322366	1.02	0.81	1.28	0.89
	Fixed-effect IVW	0.84	0.75	0.95	0.005
ADRA2B	rs4907238	1.09	0.92	1.29	0.30
	rs7559215	1.09	0.96	1.22	0.17
	rs72825850	1.09	0.99	1.21	0.09
	rs2579501	1.11	0.99	1.25	0.08
	rs7561198	1.11	1.01	1.22	0.03
	Fixed-effect IVW	1.10	1.07	1.14	$1{\times}10^{-8}$
ADRB1	rs72823015	0.87	0.74	1.02	0.09
	rs68122733	0.88	0.78	0.98	0.02
	rs7076871	0.95	0.80	1.12	0.54
	rs11196597	0.95	0.83	1.10	0.49
	rs467173	0.97	0.88	1.06	0.49
	rs11196549	0.98	0.88	1.09	0.69

	rs17875473	1.02	0.90	1.16	0.75
	rs1801253	1.02	0.96	1.09	0.50
	rs1411407	1.03	0.88	1.22	0.67
	Fixed-effect IVW	0.96	0.94	0.99	0.003

Supplementary Table 7

Individual effect estimates of each SNP selected for the MR analyses showing the effect of β1 (*ADRB1*) and β2 (*ADRB2*) activity on heart failure risk. The selected genetic instruments weighted by **systolic BP** (1 mm Hg decrease) were used as proxy for the activity of β1 and β2.

**Genes**	**SNPs**	**Odds ratio**	**Low 95% CI**	**Upp 95% CI**	***P* value**
ADRB1	rs68122733	0.91	0.85	0.97	0.007
	rs460718	0.96	0.90	1.02	0.22
	rs11196549	0.96	0.90	1.02	0.23
	rs11196597	0.96	0.88	1.05	0.38
	rs17875473	0.98	0.90	1.08	0.74
	rs1801253	1.00	0.96	1.05	0.78
	Fixed-effect IVW	0.97	0.95	0.99	0.008
ADRB2	rs10491337	0.95	0.86	1.04	0.26
	rs4579247	0.95	0.87	1.04	0.31
	rs17640858	0.98	0.89	1.07	0.66
	rs994446	0.99	0.94	1.05	0.82
	Fixed-effect IVW	0.98	0.93	1.02	0.34

SNP: single nucleotide polymorphisms; MR: Mendelian randomization; BP: Blood Pressure

Supplementary Table 8

Individual effect estimates of each SNP selected for the MR analyses showing the effect of β1 (*ADRB1*) and β2 (*ADRB2*) activity on left ventricular dimensions (LVESV, LVEDV, LV mass and LVEF). The selected genetic instruments weighted by **systolic BP** (1 mm Hg decrease) were used as proxy for the activity of β1 and β2. The effect size is reported in mL for left ventricular end-systolic volume (LVESV) and left ventricular end-diastolic volume (LVEDV), in g for left ventricular (LV) mass and in percentage for left ventricular ejection fraction (LVEF).

Genes	End points (unit)	SNPs	Effect size	Low 95% CI	Upp 95% CI	*P* value
ADRB1	LVESV (mL)	rs460718	-0.08	-0.16	0.003	0.06
		rs11196597	-0.07	-0.18	0.04	0.22
		rs1801253	-0.05	-0.10	0.002	0.06
		rs68122733	-0.004	-0.09	0.08	0.92
		rs17875473	0.006	-0.11	0.12	0.92
		Fixed-effect IVW	-0.04	-0.07	-0.02	6×10^{-4}
	LVEDV (mL)	rs460718	-0.08	-0.16	0.003	0.06
		rs11196597	-0.06	-0.17	0.05	0.29
		rs1801253	-0.01	-0.06	0.04	0.61
		rs68122733	0.02	-0.06	0.10	0.67
		rs17875473	0.06	-0.05	0.17	0.31
		Fixed-effect IVW	-0.01	-0.05	0.02	0.34
	LV mass (mL)	rs460718	-0.07	-0.16	0.007	0.07
		rs1801253	-0.04	-0.09	0.01	0.11
		rs17875473	-0.02	-0.14	0.09	0.72
		rs11196597	-0.002	-0.11	0.11	0.96

		rs68122733	0.07	-0.01	0.15	0.11
		Fixed-effect IVW	-0.02	-0.05	0.01	0.28
	LVEF (%)	rs460718	0.03	-0.05	0.12	0.41
		rs11196597	0.04	-0.07	0.15	0.46
		rs17875473	0.04	-0.07	0.16	0.48
		rs68122733	0.04	-0.04	0.13	0.32
		rs1801253	0.07	0.02	0.12	0.01
		Fixed-effect IVW	0.05	0.03	0.08	2×10^{-5}
ADRB2	LVESV (mL)	rs17640858	-0.07	-0.18	0.04	0.20
		rs4579247	-0.02	-0.14	0.09	0.68
		rs994446	0.03	-0.05	0.10	0.49
		Fixed-effect IVW	-0.005	-0.07	0.06	0.89
	LVEDV (mL)	rs17640858	-0.07	-0.18	0.04	0.19
		rs4579247	-0.04	-0.15	0.07	0.47
		rs994446	0.03	-0.05	0.10	0.48
		Fixed-effect IVW	-0.07	-0.07	0.06	0.82
	LV mass (g)	rs17640858	-0.05	-0.16	0.06	0.38
		rs4579247	-0.04	-0.15	0.07	0.48
		rs994446	0.06	-0.01	0.14	0.10
		Fixed-effect IVW	0.02	-0.05	0.09	0.55
	LVEF (%)	rs994446	-0.02	-0.10	0.05	0.59

		rs4579247	-0.01	-0.13	0.10	0.82
		rs17640858	0.03	-0.08	0.14	0.55
		Fixed-effect IVW	-0.007	-0.07	0.06	0.83

SNP: single nucleotide polymorphisms; MR: Mendelian randomization; BP: Blood Pressure

Supplementary Table 9

Individual effect estimates of each SNP selected for the MR analyses showing the effect of β1 (*ADRB1*) activity on coronary artery disease risk. The selected genetic instruments weighted by **systolic BP** (1 mm Hg decrease) were used as proxy for the activity of β1.

**Genes**	**SNPs**	**Odds ratio**	**Low 95% CI**	**Upp 95% CI**	***P* value**
ADRB1	rs17875473	0.90	0.80	1.01	0.07
	rs11196549	0.91	0.84	0.98	0.01
	rs460718	0.95	0.88	1.02	0.14
	rs68122733	0.96	0.89	1.03	0.29
	rs1801253	0.97	0.93	1.01	0.17
	rs11196597	0.97	0.89	1.06	0.52
	Fixed-effect IVW	0.96	0.94	0.98	4×10^{-5}

SNP: single nucleotide polymorphisms; MR: Mendelian randomization; BP: Blood Pressure

Supplementary Table 10

Individual effect estimates of each SNP selected for the MR analyses showing the effect of β1 (*ADRB1*) activity on heart failure risk adjusted for coronary artery disease risk. The selected genetic instruments weighted by **systolic BP** (1 mm Hg decrease) were used as proxy for the activity of β1.

**Gene**	**SNPs**	**Odds ratio**	**Low 95% CI**	**Upp 95% CI**	***P* value**
ADRB1	rs68122733	0.92	0.86	0.99	0.02
	rs11196597	0.97	0.89	1.06	0.49
	rs460718	0.97	0.91	1.04	0.46
	rs11196549	0.99	0.92	1.06	0.69
	rs1801253	1.01	0.97	1.06	0.50
	rs17875473	1.01	0.92	1.12	0.75
	Fixed-effect IVW	0.99	0.97	1.01	0.19

SNP: single nucleotide polymorphisms; MR: Mendelian randomization; BP: Blood Pressure

Supplementary Table 11

MR estimates of the effect of ADRA1A and ADRB2 expression in heart left ventricle (LV) on heart failure risk. The two selected genetic instruments were heart LV eQTLs of ADRA1A and ADRB2, respectively. The Wald estimator method was used to calculate the MR estimates with the corresponding SNP.

**Genes**	**SNPs**	**β**	**Low 95% CI**	**Upp 95% CI**	***P* value**
ADRA1A	rs7833391	0.134	-0.009	0.278	0.065
ADRB2	rs1036175	-0.051	-0.158	0.057	0.356
